# Amphiphilic Azulene-Based Fluorescent Probe for Simultaneous Monitoring of Fluctuations in Carboxylesterase Activity in Diverse Biological Samples from a Single Organism

**DOI:** 10.1021/acs.analchem.4c04926

**Published:** 2024-11-25

**Authors:** Zhenhui Cui, Yafu Wang, Ge Wang, Beidou Feng, Simon E. Lewis, Kui Wang, Kai Jiang, Tony D. James, Hua Zhang

**Affiliations:** † Collaborative Innovation Centre of Henan Province for Green Manufacturing of Fine Chemicals; Key Laboratory of Green Chemical Media and Reactions, Ministry of Education; Henan International Joint Laboratory of Smart Molecules and Identification and Diagnostic Functions; School of Chemistry and Chemical Engineering, Henan Normal University, Xinxiang, Henan 453007, P. R. China; ‡ Department of Chemistry, University of Bath, Bath BA2 7AY, U.K.; § Xinxiang Medical University, Xinxiang, Henan 453000, P. R. China

## Abstract

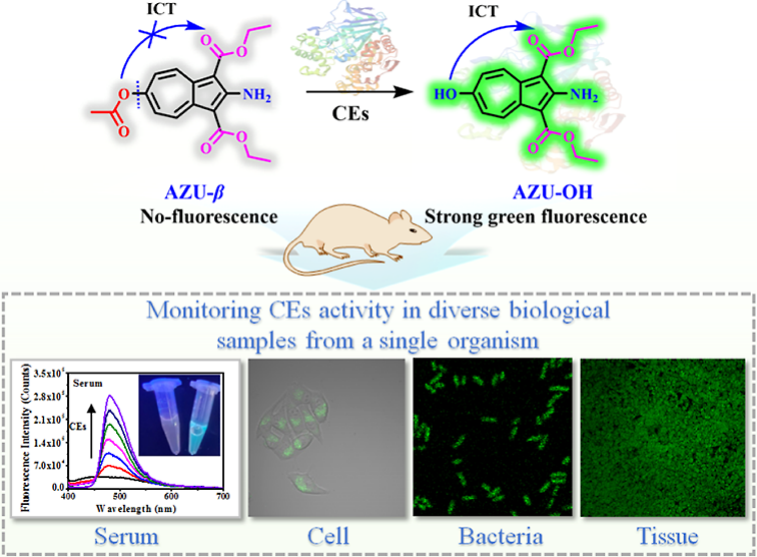

Carboxylesterase (CEs), as a key enzyme in ester metabolism, is simultaneously found with varying expression levels in diverse biological samples from a single organism, such as tissues, cells, bacteria and blood. However, the lack of integrated universal tools for the comprehensive detection of CEs’activity fluctuations in diverse biological samples from a single organism severely hinders the diagnosis and treatment of related diseases. Herein, we have developed an amphiphilic fluorescent probe (AZU-β) targeted toward CEs using an azulene derivative (AZU-OH) as a fluorophore. Using a “hydroxyl protection–deprotection” strategy, AZU-β incorporates a specific recognition group (acetyl ester) that activates the intramolecular charge transfer process to regulate the recognition signal toward CEs. AZU-β exhibits selectivity and highly sensitivity (the minimum detection limit is 1.8 × 10^–2^ U/mL), as well as rapid response (within approximately 6.0 s), for detecting CEs’activity over a wide range from 1.8 × 10^–2^ U/mL to 1.0 U/mL. Moreover, AZU-β exhibits outstanding water–oil amphiphilicity which makes it suitable for different biomembrane permeability levels. Therefore, AZU-β serves as an integrated universal tool that can not only detect CEs’activity at the serum level but also at cellular, tissue and bacterial levels under drug-induced liver injury conditions enabling the simultaneous monitoring of fluctuations in diverse biological samples from a single organism. It is expected that more probes targeting various disease-associated enzymes can be designed based on this amphiphilic design strategy to monitor relevant enzyme activity fluctuations in diverse biological samples from a single organism providing advanced analytical tools for related pathological research and diagnosis.

## Introduction

Carboxylesterases (CEs) that are important members of the esterase family, play a crucial role in many physiological processes by catalyzing the hydrolytic metabolism of many endogenous and exogenous ester substances,^1−5^ such as lipid transport and metabolism, activation and hydrolytic metabolism of ester drugs (e.g., cocaine and clopidogrel), detoxification of environmental toxicants, among others.^6−9^ These ensure that the active expression of CEs is closely related to many diseases like drug-induced liver injury (DILI), obesity, diabetes, hypercholesterolemia and various malignant tumors.^10−12^ Additionally, the expression of CEs activity is significantly different in different diseases. For example, CEs activity is down-regulated in drug-induced liver injury, while it is up-regulated in cancer.^13^ Therefore, CEs can be used as an effective and universal biological target for the diagnosis and treatment of a variety of clinical diseases. It is well-known that the occurrence and development of each disease is not independent, but interrelated, involving different organs and diverse biological samples (such as, tissues, cells, saliva, urine and blood) in different pathological stages. More importantly, different levels of CEs are simultaneously found in a diverse range of biological samples from a single organism under different diseases, which are closely related and affect the occurrence and development of different diseases. This further increases the complexity of the diagnosis and treatment of CEs-related diseases, and puts forward higher requirements for CEs detection methods in terms of selectivity, sensitivity and membrane permeability. Therefore, it becomes particularly important to develop efficient comprehensive exploration technologies for the simultaneous monitoring of CEs activity in diverse biological samples from a single organism, such as cells, bacteria, tissues, blood, etc. This holds great significance for studying related diseases’ pathology diagnosis and treatment.

At present, a variety of detection techniques, including chromatography, protein recombination, protein immunoblotting and fluorescence imaging have been reported for the detection of CEs activity.^5,14^ Among them, fluorescence imaging technologies with fluorescent probes as the core have become one of the most important techniques to detect CEs activity in complex biological environments due to the high selectivity, sensitivity, spatial and temporal resolution and negligible biological toxicity.^15−17^ As such research has focused on designing fluorescent probes for monitoring CEs activity changes in different pathological environments.^18−22^ For example, a dimethylcarbamoyl ester was introduced into the hemicyanine structure to detect the fluctuation of CEs activity during the treatment of diabetic patients under the specific enzymatic hydrolysis of CEs;^14^ while a near-infrared CEs fluorescent probe constructed with a self-immolative linker and a carbamate as recognition group was successfully used for in situ tracing of CEs activity in DILI;^11^ based on the probe design strategy of “hydroxyl protection–deprotection”, the highly sensitive and specific detection of CEs was realized, and was successfully used for the diagnosis and surgical guidance of clinical liver cancer.^23^ The above probes exhibited excellent performance for the detection of CEs in different pathological environments, respectively. They are also promising tools for the diagnosis and treatment of related diseases, as well as providing important guidance for further optimization of the performance of CEs probes. However, unfortunately, “integrated” universal probes for the detection of CEs in a variety of environments, especially those containing bacteria, are still scarce, which limits the progress toward understanding relevant pathological mechanisms and clinical treatments. Therefore, the construction of a novel fluorescent probe for the comprehensive detection of CEs in diversified biotic environments from a single organism, including bacteria, to provide a potential universal and “integrated” tool for the diagnosis and treatment of CEs-related diseases is urgently required.

For the research status and demand mentioned above, we report on an amphiphilic two-photon CEs fluorescent probe (AZU-β). In its free state, the fluorescence of AZU-β was quenched based on the “hydroxyl protection–deprotection” strategy. When combined with CEs, it could quickly and specifically respond and release the hydroxyl group, thereby enhancing the intramolecular charge transfer (ICT) process resulting in the emission of fluorescence. Additionally, AZU-β exhibited appropriate membrane permeability. Combined with the above highly sensitive “off–on” fluorescence response signal, it exhibits excellent biological properties and the potential to simultaneously detect CEs activity in diverse biological samples from a single organism. While additional research has illustrated that AZU-β could not only detect the activity changes of CEs in diverse biological samples from a single organism such as tissues, cells, blood and bacteria with high specificity and sensitivity, but can also be successfully used for the integrated detection and analysis of CEs activity changes in different pathological environments with obvious differences in activity, such as tumors and DILI. These experimental findings illustrate that AZU-β could “simultaneously” image the changes in CEs activity in diverse biological environments, and as such is expected to become a universal and “integrated” tool for CEs activity detection to promote the diagnosis and treatment of CEs-related diseases.

## Experimental Section

### Spectrographic Response of AZU-β for CEs Activity In Vitro

CEs (0–1.0 U/mL) was sequentially added to AZU-β (6.0 μM) in PBS (pH = 7.4, 37 °C). Then the test solution was mixed and evaluated immediately using an ultraviolet absorption spectrophotometer (GBC Scientific Equipment Pty LTD, Australia) and fluorescence spectrophotometer (FS5, Edinburgh Instruments, UK). In all spectral experiments, the final solutions contained <5‰ DMSO. All the experimental results were obtained from 3 parallel experiments.

### Imaging of CEs Activity Fluctuations in Diverse Biological Samples from a Single Organism under DILI

#### Fluorescence Detection in Mouse Serum

The blood of mice in control group, DILI group and treatment group was obtained, and the corresponding serum was obtained by centrifugation. AZU-β (20 μM) was added to the above three groups of serum and their fluorescence emission spectra were determined to evaluate the detection performance for CEs activity in blood. The results were obtained from 3 parallel experiments (*n* = 3).

#### Fluorescence Imaging in Tissues

Tissue sections with a thickness of 50 μm were obtained by embedding and freezing sections and placed on glass slides. Then, AZU-β (20 μM) was added to the liver, kidney, and intestinal tissue areas to ensure that the entire tissue section was always covered by the solution. The sections were incubated separately at 37 °C for 8.0 h, respectively. Then washed with PBS (pH = 7.4) three times and sealed. Fluorescence imaging was then performed under two-photon excitation at 800 nm (scan range = 495–540 nm). The results were obtained from 3 parallel experiments.

#### Fluorescence Imaging of Intestinal Bacteria in Mice

The feces of the three groups of mice were collected and placed in beef extract peptone medium, incubated on a constant temperature shaking table at 37 °C for 9.0 h. Then the samples were centrifuged at 5000 rpm for 5.0 min to obtain the following four groups of living intestinal bacteria: blank bacteria (only bacteria), control group, DILI group and treatment group. Then bacteria in the control group, DILI group and treatment group were incubated with AZU-β (20 μM) for 8.0 h, respectively. The above four groups of bacteria were imaged under 800 nm two-photon excitation to verify the specific imaging ability of AZU-β for CEs activity in living intestinal bacteria (scan range = 495–540 nm). The results were obtained from 3 parallel experiments.

## Results and Discussion

### Molecular Design

**Figure 1:**
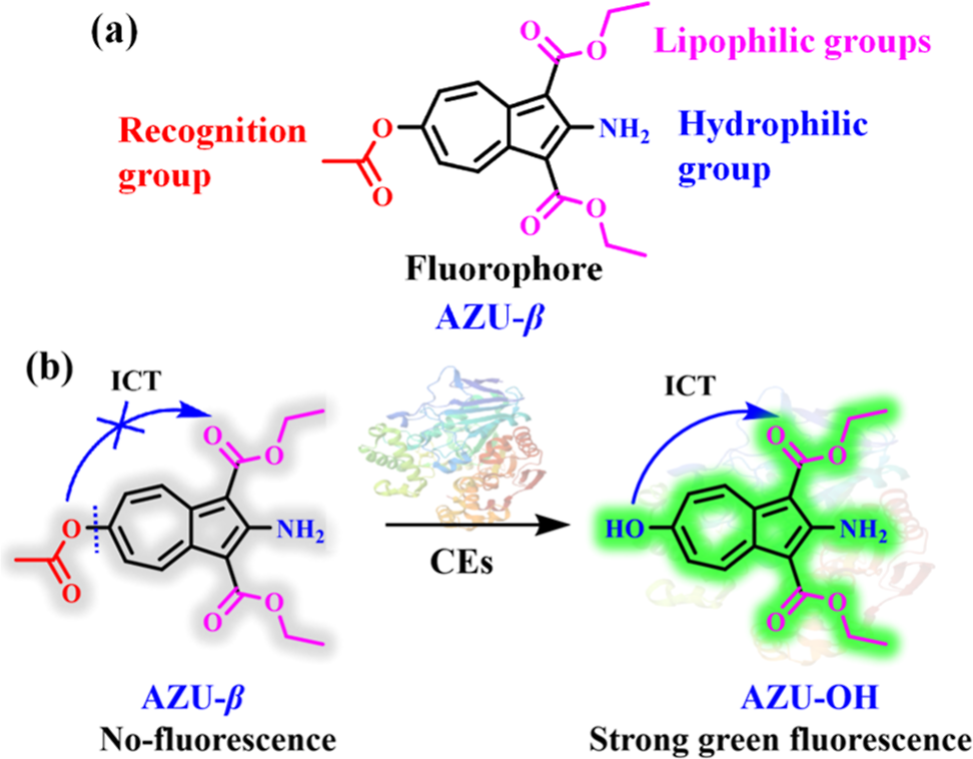
Design (a) and recognition mechanism (b) of AZU-β for CEs.

The simultaneous and comprehensive detection of carboxylesterase activity fluctuations in diverse biological samples from a single organism requires three important factors to be considered in the structural design of fluorescent probes: appropriate membrane permeability, specific and highly sensitive recognition performance for CEs. Therefore, an azulene derivative (AZU-OH) with hydrophilic groups (hydroxyl and amino) and lipophilic groups (ethyl esters) was initially selected as the fluorophore to construct the amphiphilic CEs fluorescent probe AZU-β.^24^ The azulene fluorophore has been used only rarely in biological imaging.^25,26^ However, the excellent amphiphilicity of AZU-OH provides suitable membrane permeability, allowing it to simultaneously pass through cell membranes and bacterial membranes in biological environments, which is crucial for detecting enzyme activity in diverse biological samples from a single organism.^27^ Additionally, the strong intramolecular electron push–pull system of the azulene core and enhanced ICT characteristics of AZU-OH are expected to improve the recognition sensitivity of AZU-β for CEs, thereby expanding its detection range across diverse biological samples from a single organism where CEs activity is differentially expressed. Moreover, the good structural stability and excellent two-photon properties of AZU-OH also contribute to detecting enzyme activity in diverse biological samples from a single organism. Furthermore, an acetyl ester, as a CEs-specific recognition group, was introduced into the probe structure by reacting acetyl chloride with the hydroxyl group at six-position of AZU-OH to optimize its CEs-specific recognition performance. This avoids interference from other coexisting species and facilitates the accurate detection of fluctuations in CEs activity in a single organism. This follows the common probe design strategy of CEs known as “hydroxyl protection–deprotection”, reducing the ICT characteristics of AZU-β and as expected resulting in fluorescence quenching. Therefore, only under the action of CEs hydrolysis, will the hydroxyl protection in AZU-β will be removed, and its ICT process will be restored, resulting in the generation of strong fluorescence signals resulting in specific and highly sensitive detection of CEs activity fluctuations in diverse biological samples from a single organism. The structure of AZU-β and its recognition mechanism with CEs are shown in Figure 1, while its synthetic route (Scheme S1) and structural characterization (^1^H NMR and ^13^C NMR spectra, Figures S7–S18) are presented in the Supporting Information.

### Spectral Response toward CEs Activity

**Figure 2:**
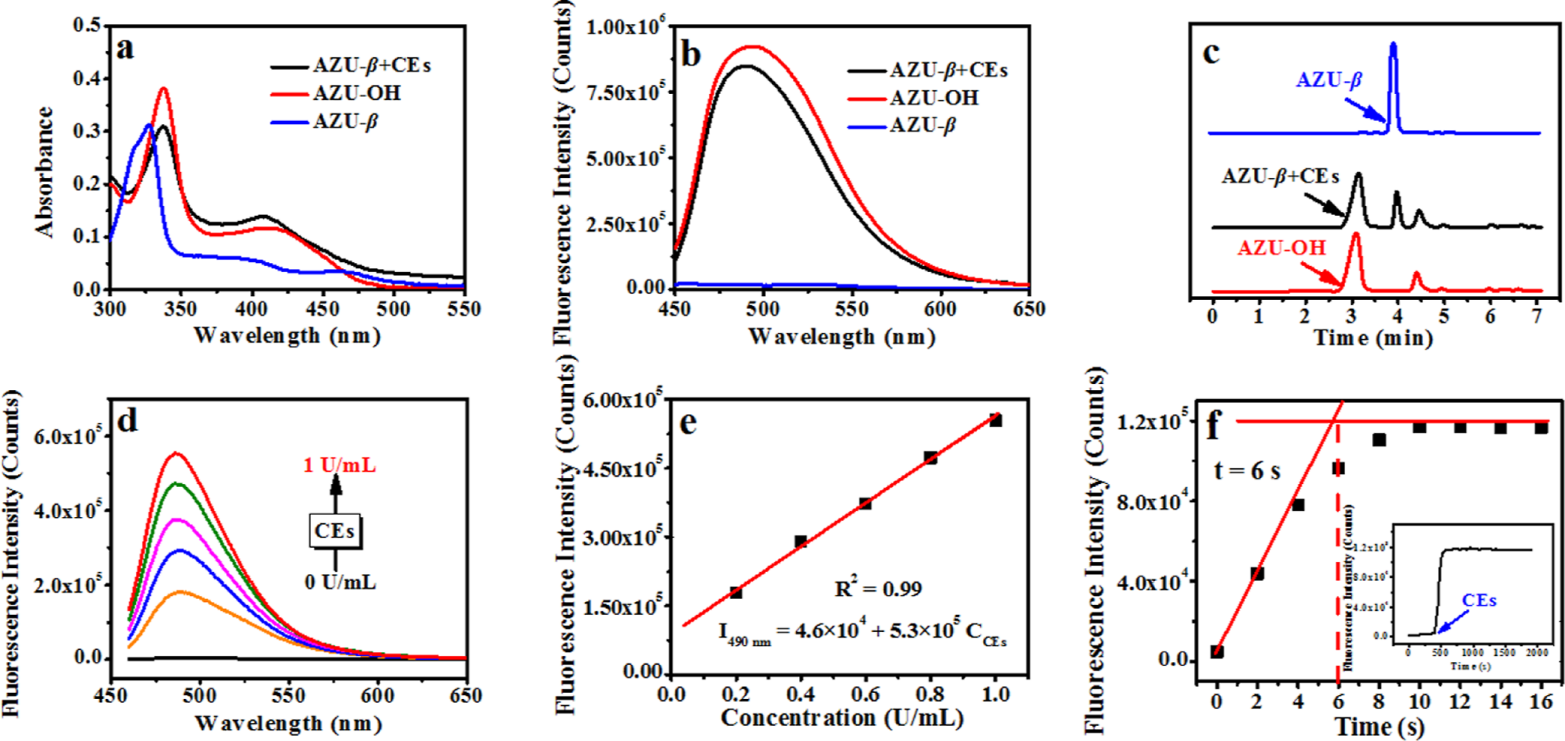
Spectral response of AZU-β (6.0 μM) for CEs in PBS (pH = 7.4, 37 °C). (a,b) Absorption (a) and fluorescence emission (b, λ_ex_ = 325 nm) spectra of AZU-β for CEs (1.0 U/mL). (c) The HPLC of AZU-β for CEs (1.0 U/mL). (d) The emission spectra of AZU-β for CEs (λ_ex_ = 325 nm, 0–1.0 U/mL). (e) Linear response of AZU-β for CEs in the emission spectrum (d), *I*_490nm_ = 4.6 × 10^4^ + 5.3 × 10^5^ C_CEs_ (*R*^2^ = 0.99). (f) Dynamic response of AZU-β for CEs (1.0 U/mL).

The spectral detection ability of AZU-β for CEs was investigated in solution (pH = 7.4 of PBS, 37 °C). The results (Figure 2) indicated that AZU-β (6.0 μM) in the free state exhibited a strong absorption peak at 325 nm (ε_AZU-β_ = 5.1 × 10^4^ M^–1^·cm^–1^), but exhibited negligible fluorescence signals (Φ_AZU-β_ = 1.4 × 10^–4^). However, when it encountered CEs (1.0 U/mL), the maximum absorption peak red-shifted to 340 nm and a strong green fluorescence signal appeared at 490 nm (enhanced ∼176-fold). These “off–on” fluorescence response results were attributed to the hydrolysis of the ester group at six-position of AZU-β (*m*/*z*_[M+Na^+^]_: 368.1119) by CEs, leading to the formation of AZU-OH (*m*/*z*_[M+Na^+^]_: 326.1003, Figure S1). This restored the strong electron-donating ability of the hydroxyl group and enhanced the ICT effect, resulting in identical spectral signals as AZU-OH (Figure 2a,b). Furthermore, this recognition process was further verified by high performance liquid chromatography (HPLC, Figure 2c). Which exhibited a retention peak at 4.0 min belonging to AZU-β which decreased significantly while a strong retention peak at 3.0 min belonging to AZU-OH appeared. Additionally, the response of AZU-β to CEs exhibited activity dependence, and the fluorescence signal at 490 nm was enhanced with an increase in CEs (Figure 2d). AZU-β demonstrated a good linear relationship with CEs activity (0–1.0 U/mL, Figure 2e) and has a minimum detection limit of 1.8 × 10^–2^ U/mL. Notably, the response time was remarkably fast as the fluorescence signal intensity reached its maximum within ∼6.0 s and remained stable thereafter (Figure 2f). This feature enables real-time monitoring of fluctuations in CEs activity under different conditions. Furthermore, Figure S2 indicated that the fluorescence response is specific for CEs and unaffected by other biological coexisting substances in cells, such as ions (Na^+^, K^+^, Cl^–^, etc.), amino acids (serine, lysine, glutamic acid, etc.), enzymes (lysozyme, trypsin, pepsin, etc.) and reactive oxygen species (ROS, hypochlorous acid, hydrogen peroxide, etc.). These results demonstrate that AZU-β could be used for highly sensitive and specific determination of CEs activity in solution (pH = 7.4 of PBS, 37 °C), making it suitable for detecting changes in CEs activity in diverse biological samples from a single organism.

The excellent structural stability of the probe forms the basis for accurately detecting CEs activity in diverse biological samples from a single organism. Therefore, we evaluated the structural stability of AZU-β by observing changes in fluorescence signals under different pH (4.0–10) and illumination time (0–5.0 h, 500 W tungsten iodide lamp). The fluorescence signal of AZU-β (6.0 μM) remained almost unchanged under different acid–base conditions (pH = 4.0–10, Figure S3a) or strong light irradiation during 5.0 h (pH = 7.4 of PBS, Figure S3b), indicating that AZU-β had high structural stability and can detect fluctuations in CEs activity without being affected by different pH levels or external light sources in diverse biological samples from a single organism. It is worth noting that the solubility of AZU-β and its metabolite AZU-OH in water was approximately 6.0 and 10 μM, respectively (Figure S4). This suggests that AZU-β is expected to have appropriate membrane permeability and be able to penetrate different cell membranes including bacterial membranes simultaneously to detect fluctuations in CEs activity in diverse biological samples from a single organism. Furthermore, negligible biotoxicity is a prerequisite for detecting CEs in diverse biological samples from a single organism. Thus, we investigated the cytotoxicity of both AZU-β and its metabolite AZU-OH using human hepatocellular carcinomas (HepG2 cells) and mouse breast cancer (4T1 cells) as models through MTT method (Figure S5). After coincubation for 24 h at a concentration of 15 μM, the survival rates of both compounds were above 90%. These results indicate that due to its extremely low biotoxicity, AZU-β can be used effectively for detecting CEs in diverse biological samples from a single organism.

### Detection of CEs Activity Fluctuations at Different Sample Levels: Serum, Cells and Bacteria

**Figure 3:**
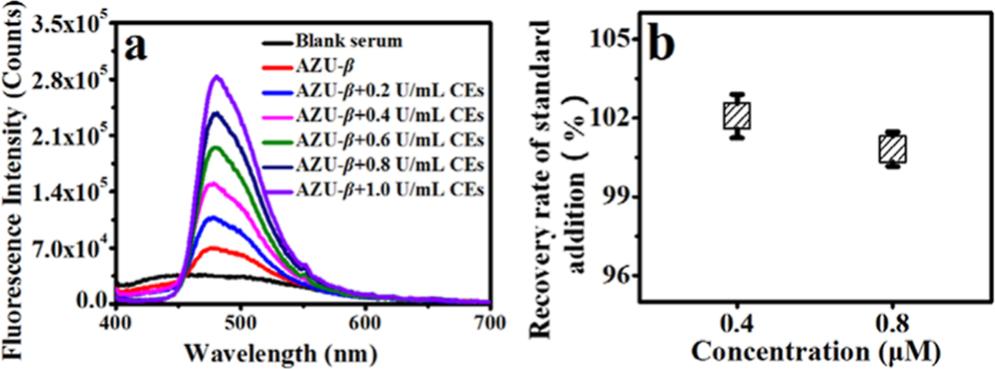
Spectral response of AZU-β (6.0 μM) for CEs in serum. (a) Fluorescence emission (λ_ex_ = 325 nm) spectra of AZU-β for CEs. (b) The spiked recoveries in (a). Blank serum: serum only without AZU-β.

Serum and urine are the most common and convenient samples for clinical disease detection, and are also the closest to the PBS buffer solution (pH = 7.4) test environment. Therefore, we first obtained serum samples from healthy contributors and used AZU-β to assess the CEs activity. The results were consistent with thatin PBS, when AZU-β was added to the serum, it quickly showed an obvious green fluorescence signal at 490 nm (Figure 3). In addition, the content of CEs was continuously increased to the above serum test system to further evaluate its detection performance for CEs activity in serum. As shown in Figure 3, with the increase of CEs activity, the fluorescence signal intensity at 490 nm was also linearly enhanced as in PBS. And based on the principle of spiked recovery experiment, using the linear relationship between CEs activity and fluorescence signal intensity at 490 nm measured in PBS, the spiked recovery rate was further calculated to be close to 100%. These results indicated that AZU-β could be used for rapid, highly sensitive detection of CEs activity in serum, which is extremely beneficial for the rapid diagnosis of clinically relevant diseases.

**Figure 4:**
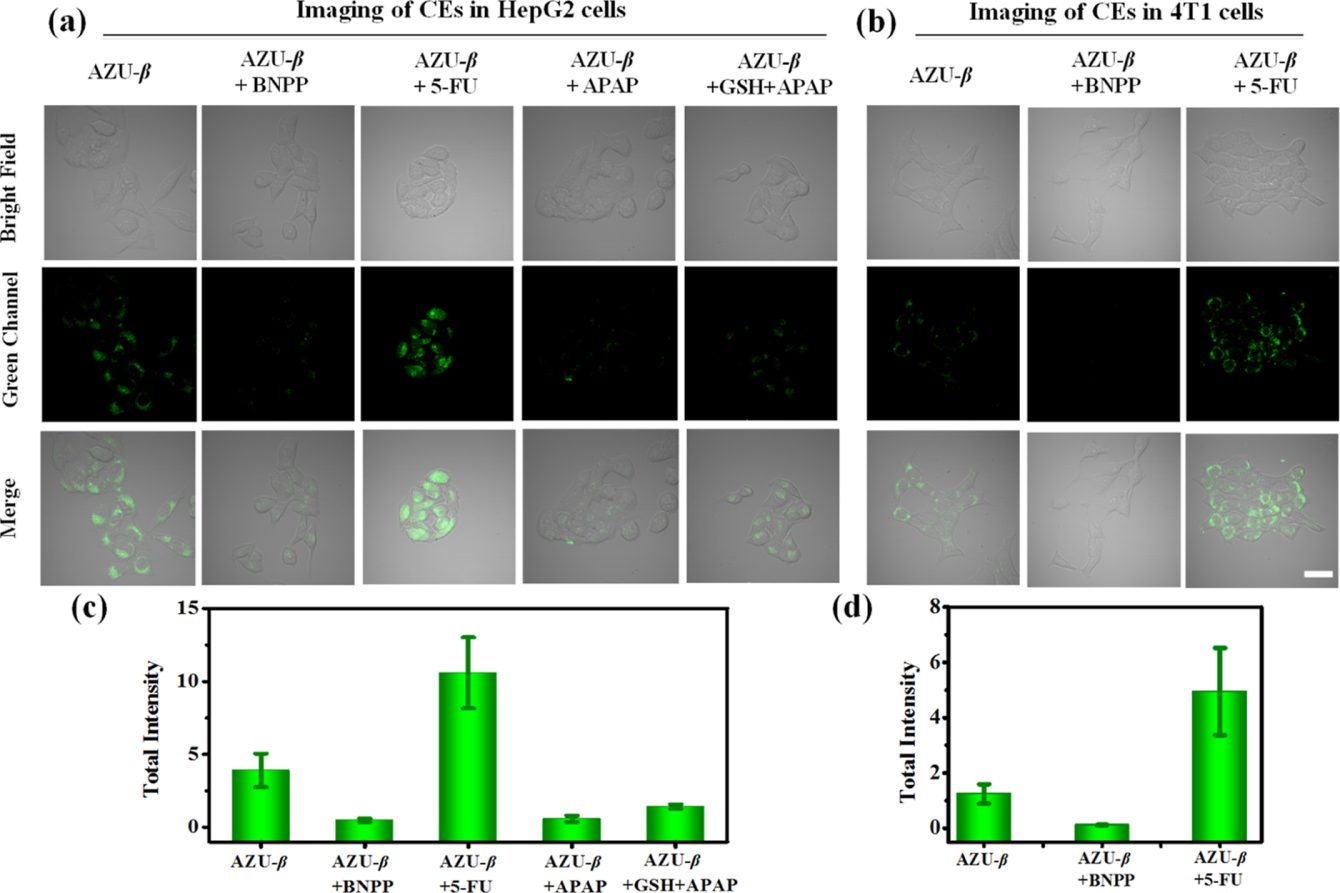
Two-photon fluorescence imaging of AZU-β (20 μM) in cancer cells (HepG2 and 4T1 cells). (a,b) Fluorescence imaging in HepG2 (a) and 4T1 (b) cells. (c,d) The total intensity data for green channels in (a) (c) and (b) (d). AZU-β group: cells were incubated with only AZU-β; AZU-β + BNPP group: cells were incubated with BNPP (200 μM) for 30 min, and then incubated with AZU-β; AZU-β + 5-FU group: cells were pretreated with 5-FU (20 μM) for 2.0 h, and then incubated with AZU-β. AZU-β + APAP group: cells were pretreated with APAP (1.0 mM) for 2.0 h, and then incubated with AZU-β. AZU-β + GSH + APAP group: cells were pretreated with GSH (1.0 mM) for 2.0 h, then incubated with APAP (1.0 mM) for 2.0 h, and finally incubated with AZU-β. Two-photon excitation wavelength = 800 nm, scan range of green channel = 495–540 nm. Internal PMTs are at 16 bit and 1600 × 1600 pixels, and scan speed is 400 Hz. Scale bar: 20 μm.

The CEs activity was found to be overexpressed in tumor cells, particularly in hepatoma cells. Therefore, HepG2 and 4T1 cells were subsequently selected as research subjects to evaluate the ability of AZU-β in detecting CEs activity at living cellular level. The results (Figure 4) demonstrated that after coincubation of AZU-β (20 μM) with HepG2 and 4T1 cells, both exhibited noticeable fluorescence signals in the green channel (495–540 nm) under an 800 nm two-photon laser excitation. To confirm that the fluorescence signal specifically responded to CEs in tumor cells, the cells were pretreated with bis(4-nitrophenyl) phosphate (BNPP), a covalent inhibitor of CEs, at a concentration of 200 μM for 30 min. It could be clearly observed that the fluorescence signal intensity was significantly reduced or negligible due to inhibition of the CEs activity by BNPP. In contrast, it is worth noting that when the cells were pretreated with fluorouracil (5-FU), an inducer of CEs at a concentration of 20 μM for 2.0 h, the fluorescence signal intensity was significantly enhanced compared to untreated cells. Furthermore, the fluorescence signal intensity was notably stronger in HepG2 cells than in 4T1 cells, which is consistent with previous reports indicating higher expression levels of CEs activity in liver cancer compared to breast cancer cell lines.^28,29^ These results demonstrated that changes observed in fluorescence signals are indeed caused by alterations in CEs activity within tumor cells. On this basis, HepG2 cells were pretreated with acetaminophen (APAP, 1.0 mM, a drug that causes DILI) to down-regulate the CEs activity. The results showed that the fluorescence signal of APAP-stimulated cells was significantly lower than that of untreated cells, which was almost negligible. Meanwhile, when cells were pretreated with glutathione (GSH, 1.0 mM, a substance that alleviates DILI) for 2.0 h before adding APAP (1.0 mM), the fluorescence signal intensity in the green channel was significantly stronger than that observed in cells treated with APAP alone. This was mainly due to the downregulation of CEs activity expression in cells by APAP pretreatment, resulting in many hydroxyl groups of AZU-β remaining in a protected state, and leading to unrecoverable fluorescence. However, GSH can effectively alleviate the effect of APAP, and its CEs activity is significantly upregulated compared with that of APAP-stimulated cells, thus exhibiting significantly enhanced fluorescence signals. These results indicated that AZU-β can specifically and highly sensitively monitor the fluctuation of CEs activity in cells and even tumor cells.

The above results confirmed the excellent detection performance of AZU-β at the serum and cell level. However, in addition to serum and cells, CEs has also been reported to be overexpressed in various bacteria, such as *Escherichia coli* and *Staphylococcus aureus*.^30^ Nevertheless, most existing probes face difficulties in penetrating bacterial membranes to detect fluctuations in their CEs activity. Thus, *E. coli* (Gram-negative) and *S. aureus* (Gram-positive) were selected as research subjects and incubated with AZU-β for imaging purposes. The results demonstrate that both bacteria exhibit noticeable fluorescence signal in the green channel (Figure S6). Moreover, after pretreatment with BNPP (200 μM), their fluorescence signal intensity decreased significantly. This confirmed our initial hypothesis that due to the simultaneous presence of hydrophilic and lipophilic groups in its molecular structure, AZU-β can penetrate not only cell membranes but also bacterial membranes at a living level while enabling specific and highly sensitive imaging of CEs activity within the bacteria (both Gram-negative and Gram-positive). This finding not only contributes to the diagnosis and treatment of diseases caused by bacteria but also expands the scope of application of AZU-β.

### Imaging of CEs Activity Fluctuations in Diverse Biological Samples from a Single Organism under DILI

**Figure 5:**
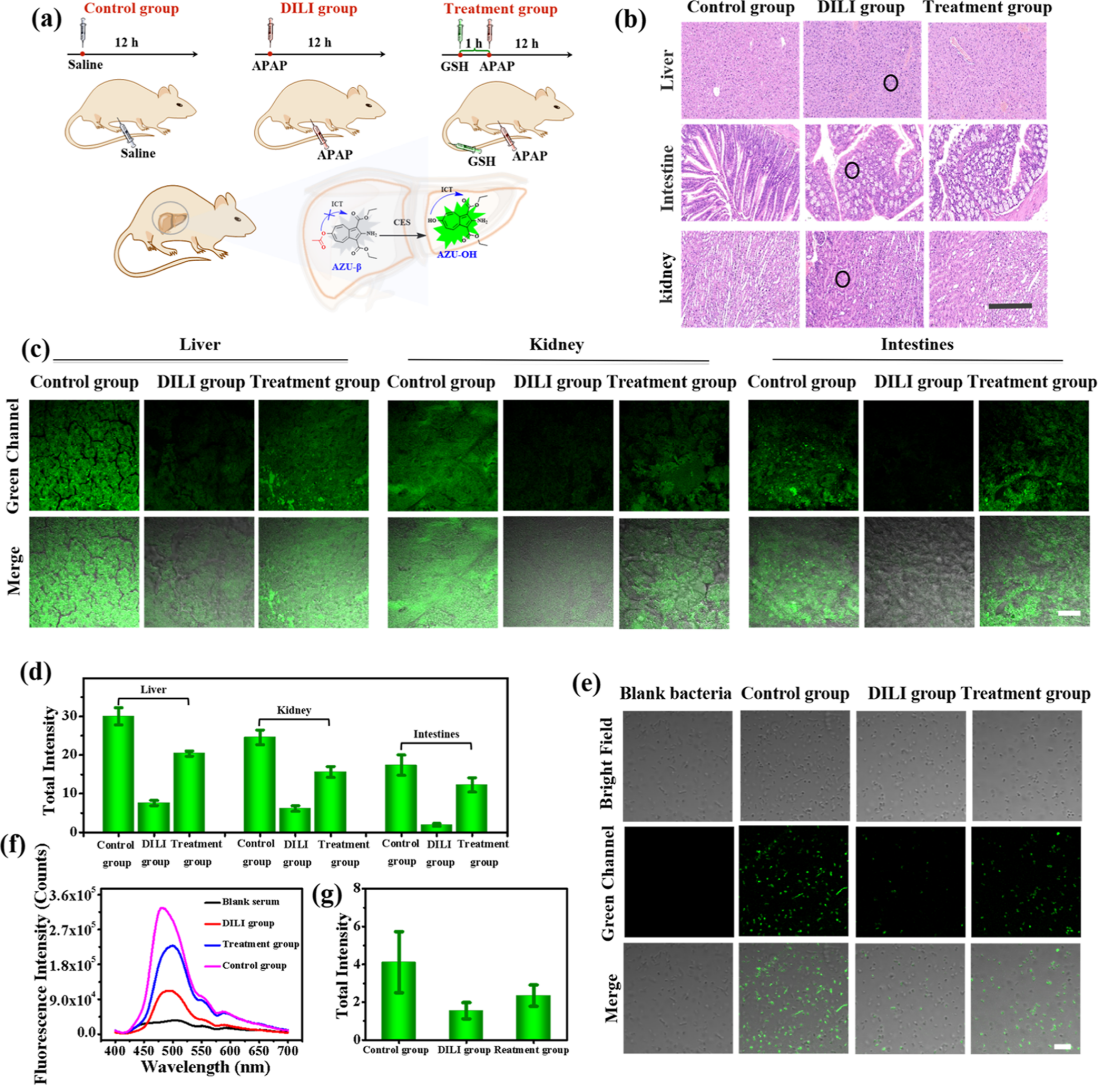
Two-photon fluorescence imaging of AZU-β (20 μM) for CEs in diverse biological samples from a single organism under DILI. Two-photon fluorescence imaging of AZU-β (20 μM) for CEs in living tissue with DILI. (a) Construction of DILI mouse model. (b) H&E staining analysis the injury degree of liver, kidney and intestinal tissue. Scale bar: 40 μm. (c) Fluorescence imaging in tissue with DILI. (d) The total intensity data for green channels in (c). (e) Fluorescence imaging in bacteria. (f) Fluorescence emission (λ_ex_ = 325 nm) spectra of AZU-β for CEs in serum. (g) The total intensity data for green channels in (e). DILI mouse model in a, control group: mouse were intraperitoneally injected with normal saline; DILI group: mouse were intraperitoneally injected with APAP (400 mg/kg) for 12 h; treatment group: mouse were injected with GSH (200 mg/kg) through the tail vein for 1.0 h, followed by intraperitoneal injection of APAP (400 mg/kg) for 12 h. Then, the tissues with different degrees of damage were incubated with AZU-β respectively for imaging. Mouse gut bacteria were obtained through mouse feces and imaged. The blank bacteria were pure bacteria that were not coincubated with AZU-β. Two-photon excitation wavelength = 800 nm, scan range = 495–540 nm. Incubation concentration: 20 μM. Incubation time: 8.0 h. Internal PMTs are at 16 bit and 1600 × 1600 pixels, and scan speed is 400 Hz. Scale bar: 20 μm.

Inspired by the excellent properties of AZU-β, further exploration was conducted to investigate its biological applications in diverse biological samples from a single organism. Kunming mice (KM) were selected as the research object, while DILI was used as the pathological model. A DILI mouse model was established by injecting a high dose of acetaminophen (APAP) to evaluate its ability to detect changes in CEs activity before and after DILI treatment in vivo.^31−34^ The DILI mouse models were divided into three groups as shown in Figure 5a: (1) the control group received an intraperitoneal injection of normal saline; (2) the DILI experimental group (APAP) received an intraperitoneal injection of APAP (400 mg/kg) for 12 h; (3) the DILI treatment group (APAP + GSH) received a tail vein injection of GSH (200 mg/kg), followed by an intraperitoneal injection of APAP (400 mg/kg) for 12 h. Hematoxylin–eosin staining (H&E staining) was used to analyze the degree of injury of liver, kidney and intestinal tissue. As shown in Figure 5b, compared with the control group, the tissues treated with APAP exhibited obvious injury, such as (black oval mark) spotty necrosis in liver tissue, serious disorder in intestinal villi size ratio, increased goblet cells, and renal tubule edema. And, although the APAP-GSH treatment group had the same damage phenomena, they were obviously lighter than those in the group treated with APAP only. These results confirmed successful establishment of both the DILI model and DILI treatment model. Subsequently, the serums of the three groups were obtained, and the fluorescence signal at 490 nm was rapidly enhanced after adding AZU-β (Figure 5f). Moreover, the fluorescence signal of the control group was obviously the strongest, and that of the DILI experimental group and the treatment group decreased in turn. In addition, the liver, intestine, and kidney tissues of the three groups of mice were incubated with AZU-β and imaged under an 800 nm laser by two-photon fluorescence microscopy. As expected, the tissues treated with saline in the control group showed bright fluorescent signals, whereas those treated with APAP in the DILI group exhibited extremely weak signals (Figure 5c,d). Interestingly, the tissues treated with both APAP and GSH in the treatment group exhibited significantly enhanced fluorescence signal compared to those treated with only APAP. Meanwhile, the corresponding mouse intestinalbacteria were obtained from the feces of three groups of mice and incubated with AZU-β for imaging, which exhibited the same results as tissue imaging. The fluorescence signal of the control group was significantly stronger than that of the treatment group, and the fluorescence signal of the DILI experimental group was negligible (Figure 5e,g). These results are consistent with reports that CEs activity is expressed in metabolic tissues such as liver, kidney, and intestine, with higher activity expression in liver tissues.^1,2^ These results indicated that the CEs activity of the three tissues were significantly down-regulated under DILI conditions.^7^ More importantly, it proved that AZU-β could detect the fluctuations of CEs activity in diverse biological samples from a single organism under DILI, which is expected to become a universal and “integrated” tool for CEs activity detection to promote the diagnosis and treatment of CEs-related diseases.

## Conclusions

In summary, following the “hydroxyl protection–deprotection” strategy, a CEs fluorescent probe AZU-β was constructed using an amphiphilic azulene derivative as a fluorophore and an acetyl ester as a specific recognition group. AZU-β enables rapid, specific and highly sensitive monitoring of CEs activity over a wide range from 1.8 × 10^–2^ to 1.0 U/mL to generate varying amounts of product AZU-OH. The minimum detection limit of AZU-β for CEs is 1.8 × 10^–2^ U/mL. Meanwhile, the design of an amphiphilic molecular structure allows both AZU-β and its product AZU-OH to exhibit good membrane permeability for the cytomembrane and bacterial wall. Therefore, not only can AZU-β individually detect CEs activity at serum, cell, and bacteria, but it can also simultaneously monitor fluctuations in CEs activity in diverse biological samples from a single organism under drug-induced liver injury. These characteristics make AZU-β promising as an integrated universal tool for studying the pathology and diagnosis and treatment of CEs-related diseases. It is anticipated that the “hydroxyl protection–deprotection” strategy could be employed to design more integrated universal tools for different bioenzymes in diverse biological samples from a single organism, which are associated with clinically significant malignant diseases.

## Supporting Information Available

Experimental materials, chemicals, reagents. Specific experimental methods, AZU-β synthetic route and NMR characterization. Photostability, pH stability, solubility, cytotoxicity, and other supplementary charts for AZU-β (PDF)
