# Supplementary material for: Amphiphilic Azulene-Based Fluorescent Probe for Simultaneous Monitoring of Fluctuations in Carboxylesterase Activity in Diverse Biological Samples from a Single Organism
Source: Anal Chem. 2024 Nov 25;96(49):19732–9. doi: 10.1021/acs.analchem.4c04926 (PMC11656413; doi:10.1021/acs.analchem.4c04926)
Supplement: Supplementary file 1 — ac4c04926_si_001.pdf [file ac4c04926_si_001.pdf]

Supporting Information

Amphiphilic Azulene-Based Fluorescent Probe for Simultaneous Monitoring of Fluctuations in Carboxylesterase Activity in Diverse Biological Samples from a Single Organism

Zhenhui Cui,[†,‖] Yafu Wang,[†,‖] Ge Wang,[§] Beidou Feng,[†] Simon E. Lewis,[‡] Kui Wang,[†] Kai Jiang,[†] Tony D. James[*,†,‡] and Hua Zhang[*,†]

[†] Collaborative Innovation Centre of Henan Province for Green Manufacturing of Fine Chemicals; Key Laboratory of Green Chemical Media and Reactions, Ministry of Education; Henan Key Laboratory of Organic Functional Molecule and Drug Innovation; School of Chemistry and Chemical Engineering, Henan Normal University, Xinxiang, Henan 453007, P. R. China; [‡] Department of Chemistry, University of Bath, Bath, BA2 7AY, U.K.; [§] Xinxiang Medical University, Xinxiang 453000, P. R. China; [‖] Zhenhui Cui and Yafu Wang contributed equally to this work; [*] Corresponding Author Email: zhanghua1106@163.com, t.d.james@bath.ac.uk, Phone/Fax: +86-373-3329030.

Table of Contents

1. **Experimental Procedures……S2**
2. **The synthesis and structural characterization of AZU-*β*……S5**
3. **The HRMS spectrum of AZU-*β* for CEs activity……S7**
4. **The biocompatibility of AZU-*β*……S8**
5. **The fluorescence imaging of AZU-*β* in bacteria……S9**
6. **Appendix……S10**

1. Experimental Procedures

Procedures section.

The synthesis routes of the fluorescent probe **AZU-*β*** and their intermediates are shown in Scheme S1. The reagents used in the synthesis and purification of probes and intermediates were all analytical grade. They were purified by column chromatography using silica gel (200-300 mesh). And the structures were characterized by Avance 600 MHz NMR spectrometer (Bruker Co., Switzerland) and Ultra-high-resolution electro-spray time-of-flight mass spectrometry (Germany Brooke). UV spectrophotometer (GBC Scientific Equipment Pty LTD, Australia) and fluorescence spectrometer (FS5, Edinburgh Instruments, UK) were used for the basic spectral experiments *in vitro*. The response mechanism was determined by high performance liquid chromatograph (HPLC, 1260 Infinity II, Agilent, Germany). And the biological properties were verified by microplate reader, FV1200 spectral confocal multiphoton spectrometer (Olympus, Japan). Carboxyesterase (EC 3.1.1.1.) was obtained from Sigma Chemical Co. (USA). 6.0 mM the probe solutions (**AZU-*β***) were prepared using DMSO for the optical and biological experiments.

Dynamics testing.

CEs (1.0 U/mL) was added to **AZU-*β*** (6.0 μM) test solutions (PBS, pH = 7.4). The fluorescence signal intensity of **AZU-*β*** at 490 nm was detected to verify the response time of the probe with CEs.

Response mechanism.

The retention time of **AZU-*β*** (10 μM) and AZU-OH aqueous solutions was measured in 97% methanol solution by HPLC. Then, CEs (1.0 U/mL) was added to **AZU-*β*** (10 μM) aqueous solution, and its retention peak change and corresponding retention time were tested. The corresponding recognition mechanism was determined by comparing the retention peak and retention time before and after recognition.

Selectivity.

Six biological macromolecules including Lysozyme, β-amylase, trypsin, pepsin, protease K and carboxylesterase were selected as interferences. These were added (20 times the equivalent of CEs activity) to the **AZU-*β*** (6.0 μM, λ_{ex} = 325 nm) test solutions (PBS, pH = 7.4), and the change of fluorescence intensity measured, respectively.

Similarly, the biological coexisting substances cation and anion (include $MnSO_4$, Li_2CO, $AgNO_3$, $Zn(NO_3)_2$, $MgSO_4$, $CuSO_4$, $FeCl_2$ $4H_2O$, $SnCl_2$, $NaHSO_4$, $CaCl_2$ $6H_2O$, $COCl_2$, $FeCl_3$ $6H_2O$, $CdCl_2$, KCl, NaCl.), amino acid (include serine, lysine, Glutamine, DL-homocysteine, dithiothreitol acid, glycine-L-complex acid, D-cysteine, Glutamate, glycine, DL-threonine, Cystine, arginine, DL-leucine, L-aspartic acid, Hypoxanthine.) and reactive oxygen species (including $O_2^{\cdot-}$, $NO^{\cdot}$, H_2O_2, TBO, NaClO, $^1O_2$, TBH, $OH^{\cdot}$) were added to the test solution (PBS, pH = 7.4) at 20 equivalents to detect the change of fluorescence signal and evaluate the interference ability of CEs monitoring.

Photostability in solution.

AZU-*β* (6.0 μM) was dissolved in PBS (pH = 7.4). The solutions were irradiated by a 500 W iodine-tungsten lamp situated 250 mm away for 5.0 h. An aqueous solution of sodium nitrite (50 g/L) was placed between the samples and the lamp as a light filter (to cut off the light shorter than 400 nm) and as a heat filter. The photostabilities were expressed in terms of ratio fluorescence intensity (%) calculated from the changes of the fluorescence signal intensity at the maximum strength before

and after irradiation by iodine-tungsten lamp. The fluorescence intensity was determined (λ_{ex} = 325 nm, λ_{em} = 490 nm). The data were obtained from replicate experiments (n = 3).

pH-stability in solution.

The test solutions with different pH (pH = 3.0-10) were obtained by adjusting strong acid (HCl) and strong base (NaOH). Then the fluorescence signal intensity of **AZU-*β*** (6.0 μM, λ_{ex} = 325 nm, λ_{em} = 490 nm) at different pH test solution were measured using a fluorescence spectrometer, respectively. The changes of fluorescence intensity at different pH were analyzed, in order to determine the influence of different pH on the detection of probes to CEs activity. The data were obtained from replicate experiments (n = 3).

Solubility in water.

The probe **AZU-*β*** (1.0 μM-9.0 μM) and enzyme metabolite AZU-OH (2.0 μM-12 μM) were continuously added into H_2O, respectively. Then the absorbance of the probes at different concentrations were determined, and the solubility of **AZU-*β*** and AZU-OH in H_2O were obtained according to the Lambert Beer's law (A = εbC). The data were obtained from replicate experiments (n = 3).

Cell culture.

HepG2 (human liver cancer cell) and 4T1 (mouse breast cancer cell) were obtained from the Chinese Academy of Medical Sciences. The red-free Dulbecco's Modified Eagle's Medium (DMEM, WelGene) supplemented with penicillin/streptomycin and 10 % fetal bovine serum (FBS; Gibco) were used for culture cells in a CO_2 incubator at 37 ^{o}C. Before imaging, the cells were seeded into a glass bottomed dish (MatTek, 35 mm dish with 20 mm well) for incubation 24 h in advance at 37 ℃ under a 5% CO_2 atmosphere. And then, the cells were incubated with a certain concentration of **AZU-*β*** (20 μM) at 37 ℃ under a 5% CO_2 atmosphere, respectively.

Cytotoxicity.

HepG2 cell line and 4T1 cell line were prepared for cell viability studies in 96-well plates (1×10^5 cells per well that were incubated in 100 μL). The cells were incubated for an additional 24 h with **AZU-*β*** in different concentrations (0, 5.0, 10, 15, 20 μM). Subsequently, 20 μL 5 mg/mL of 3-(4,5-dimethylthiazol-2-yl)-2,5-diphenyltetrazolium bromide (MTT, Sigma Chemical Co. U.S.A.) was added into each well, followed by further incubation for 4 h at 37 ^{o}C under a 5% CO_2 atmosphere. The DMEM was remove and DMSO (150 μL) was added to dissolve the reddish-blue crystals. Optical density (OD) was determined by a microplate reader (Spectra Max M5, Molecular Devices) at 490 nm. The results from the six individual experiments were averaged. The relative cell viability (100%) was calculated using the following equation:

$$\text{Cell viability(\%)} = (OD_{probe}-OD_{k\text{-}probe})/(OD_{ctrl}-OD_{k\text{-}ctrl}) \times 100 \qquad (1)$$

Spectrographic response of AZU-*β* for CEs activity in serum.

The blood of cervical cancer contributors was obtained and the serum was obtained by centrifugation. **AZU-*β*** (6.0 μM) was added to the serum and its fluorescence emission spectrum was measured by fluorescence spectrophotometer (FS5, Edinburgh Instruments, UK). Then CEs (0 - 1.0 U/mL) was successively added into the serum test solution, and the change of its fluorescence emission spectrum was measured. And the recovery rate of the added standard was tested according to the experiment of added standard recovery. In all spectral experiments, the final solutions contained < 5 ‰ DMSO. All the experimental results were obtained from 3 parallel experiments.

Imaging of CEs activity in cancer cells.

Olympus spectral confocal multiphoton microscope (FV1200) with MaiTai femtosecond laser source (Spectra-Physics) was used in cell imaging. The imaging parameters are as follow. Internal PMTs = 16 bit, pixels = 1600 × 1600. Lasers: 800 nm. The scan ranges were ascertained according to fluorescence of the probe.

For CEs imaging activity experiments in different cancer cells, HepG2 and 4T1 cells were incubated with **AZU-*β*** (20 μM) for 8.0 h at 37 ^{o}C under a 5% CO_2 atmosphere, respectively. For CEs inhibition imaging experiments, HepG2 and 4T1 cells were incubated with BNPP (200 μM) for 30 min, respectively, and then the cells were incubated with **AZU-*β*** (20 μM) for 8.0 h at 37 ^{o}C under a 5% CO_2 atmosphere. For CEs-stimulated imaging experiments, HepG2 and 4T1 cells were incubated with 5-FU (20 μM) for 2.0 h, respectively, and then the cells were incubated with **AZU-*β*** (20 μM) for 8.0 h at 37 ^{o}C under a 5% CO_2 atmosphere. In APAP induction experiments, HepG2 cells were pretreated with APAP (1.0 mM) for 2.0 h and then incubated with **AZU-*β*** (20 μM) for 8.0 h at 37 ^{o}C under a 5% CO_2 atmosphere. In the APAP inhibition remission experiment, HepG2 cells were pretreated with GSH (1.0 mM) for 2.0 h, then incubated with APAP (1.0 mM) for 2.0 h, and finally incubated with **AZU-*β*** (20 μM) for 8.0 h at 37 ^{o}C under a 5% CO_2 atmosphere. The above cells were washed three times with PBS and then imaged under two-photon excitation at 800 nm to verify the specific response of **AZU-*β*** to endogenous CEs activity in cells (scan range = 495-540 nm). The data were obtained from replicate experiments (n = 3).

Imaging of CEs activity in bacteria.

E. coli and *Staphylococcus aureus* in logarithmic phase of growth were taken and divided into blank control group, probe group and inhibitor group. The volume of each group was 400 μL, and equal volumes of DMSO, probe, probe and inhibitor were added respectively. The concentration of **AZU-*β*** was 20 μM, and the concentration of inhibitor BNPP was 200 μM, incubated at 37 ℃ constant temperature shaker for 9.0 h, centrifuged at 5000 r for 5.0 min to collect the organisms, resuspended. Precipitated by sterile PBS (pH = 7.4) to clean the organisms, and centrifuged again to collect the organisms. Then imaged under two-photon excitation at 800 nm to verify the specific response of **AZU-*β*** to CEs activity in bacteria (scan range = 495-540 nm). The data were obtained from replicate experiments (n = 3).

Imaging of CEs activity fluctuations in diverse biological samples from a single organism under DILI.

In this work, all the animal experiments involved in this study have been approved by the local research ethics review committee of the Animal Ethics Committee of Xinxiang Medical College (Henan, China, ethical statement reference number 2015016). And all mices are used in accordance with the regulations and guidelines of the institutional ethics Committee on animal welfare. The mice used were Kunming mouse (KM, 5 weeks old) purchased from Beijing Weitong Lihua Experimental Animal Technology Co., Ltd.. Mice were placed in an independent ventilated cage system (IVC) and fed with distilled water and SPF feed. The mice were divided into three groups. Control group: mice were intraperitoneally injected with normal saline; DILI group: mice were intraperitoneally injected with APAP (400 mg/Kg) for 12 h; Treatment group: mice were injected with GSH (200 mg/Kg) through the tail vein for 1.0 h, followed by intraperitoneal injection of APAP (400 mg/Kg) for 12 h. Then, the blood, metabolic tissue (Liver, kidney and intestinal tissue) and feces of mice before and after DILI treatment was obtained.

H&E staining experiment.

The liver, kidney and intestine tissues of Kunming mice (KM) were fixed in 10% formaldehyde immediately after sacrifice. Histological examination was according to a conventional method, and stained with hematoxylin and eosin (H&E). Classification and recording of the morphology of any observed lesions was according to classification criteria.

2. The synthesis and structural characterization of AZU-*β*

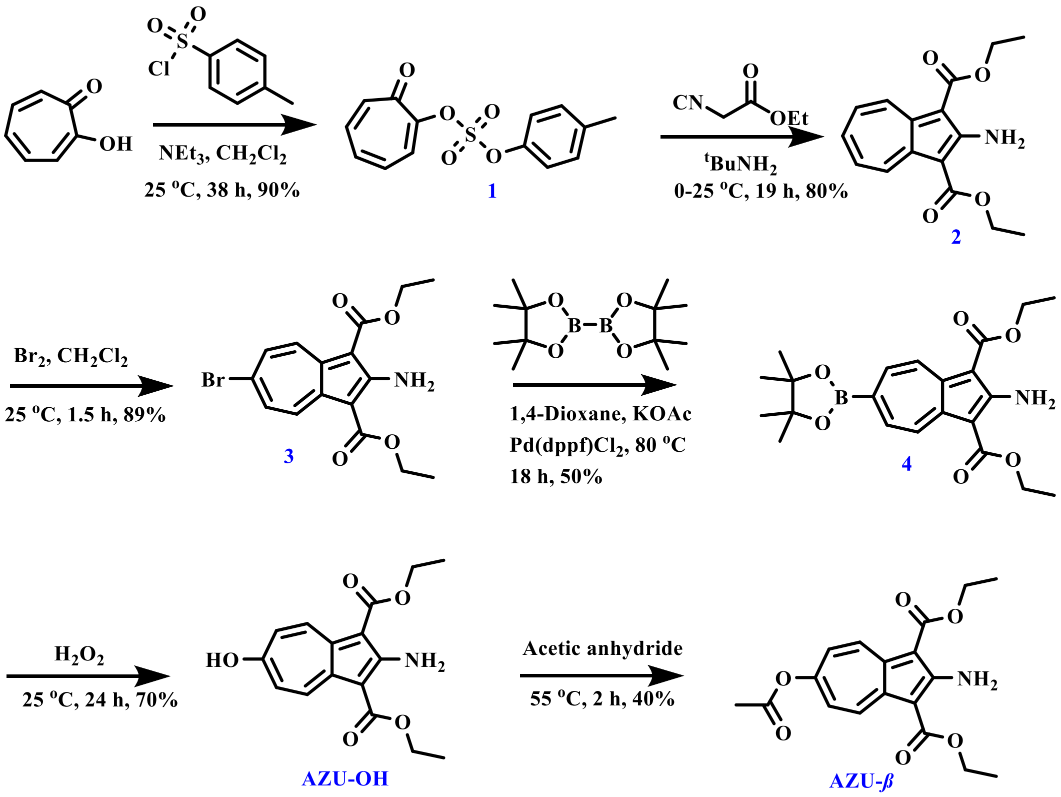

Scheme S1. The synthetic route of **AZU-*β***

The synthesis of 1 (7-Oxocyclohepta-1,3,5-trien-1-yl 4-methylbenzenesulfonate). Under an atmosphere of nitrogen, tropolone (1.00 g, 8.20 mmol, 1.00 eq) and tosyl chloride (1.56 g, 8.20 mmol, 1.00 eq) were dissolved in anhydrous CH_2Cl_2 (20 mL), to which NEt_3 (1.14 mL, 8.20 mmol, 1.00 eq) was added dropwise. The solution was further diluted in CH_2Cl_2 (12 mL) to prevent a suspension from forming, and left to stir for 38 h, affording a yellow slurry. The reaction was quenched with ice, extracted with CH_2Cl_2 (3 × 20 mL), dried with $MgSO_4$, filtered and concentrated under reduced pressure affording 7-oxocyclohepta-1,3,5-trien-1-yl 4-methylbenzenesulfonate **1** as a crystalline yellow solid. Yield 90% (2.30 g). ^{1}H NMR (600 MHz, $CDCl_3$) δ 7.93 (d, *J* = 8.2 Hz, 2H), 7.46 (d, *J* = 9.4 Hz, 1H), 7.35 (d, *J* = 8.2 Hz, 2H), 7.21 (dd, *J* = 12.3, 8.0 Hz, 1H), 7.15 (d, *J* = 12.1 Hz, 1H), 7.11 – 7.05 (m, 1H), 6.98 (t, *J* = 10.2 Hz, 1H), 2.45 (s, 3H). ^{13}C NMR (151 MHz, $CDCl_3$) δ 179.43, 155.18, 145.51, 141.26, 136.32, 134.60, 133.45, 130.83, 130.00, 129.62, 128.62, 21.78.

The synthesis of 2 (Diethyl 2-aminoazulene-1,3-dicarboxylate). 7-Oxocyclohepta-1,3,5-trien-1-yl 4-methylbenzenesulfonate **1** (1.00 g, 3.62 mmol, 1.00 eq) and ethyl cyanoacetate (0.80 mL, 8.00 mmol, 2.20 eq) were dissolved in ethanol (18 mL) and cooled to 0 ℃, to which *t*-butylamine (3.90 mL, 9.00 mmol, 2.50 eq) was added dropwise. The solution was left to stir for 19 h. During this period, the system was heated to room temperature, and the product, orange precipitate, had been formed. The precipitate was filtered, washed with water, and dried under vacuum to obtain diethyl 2-aminoazulene-1,3-dicarboxylate **2** as a bright orange solid without further purification. Yield 80% (0.80 g). ^{1}H NMR (600 MHz, $CDCl_3$) δ 9.16 (d, *J* = 10.2 Hz, 2H), 7.79 (s, 2H), 7.55 (app t, *J* = 10.2 Hz, 2H), 7.44 (t, *J* = 9.6 Hz, 1H), 4.47 (q, *J* = 7.1 Hz, 4H), 1.48 (t, *J* = 7.1 Hz, 6H). ^{13}C NMR (151 MHz, $CDCl_3$) δ 166.60, 162.49, 146.15, 132.90, 132.65, 131.43, 99.68, 59.86, 14.69.

The synthesis of 3 (Diethyl 2-amino-6-bromoazulene-1,3-dicarboxylate). Diethyl 2-aminoazulene-1,3-dicarboxylate **2** (1.00 g, 3.48 mmol, 1.00 eq) was dissolved in anhydrous CH_2Cl_2 (20 mL) and cooled to 0 ℃, to which bromine (0.20 mL,

3.83 mmol, 1.10 eq) was added over a 20 min period. The solution was warmed to room temperature and stirred for 1.5 h. The reaction was quenched with water (150 mL), separated and the aqueous layer was extracted with CH_2Cl_2 (2 × 50 mL). The collected organic extracts were dried with $MgSO_4$, filtered and concentrated under reduced pressure. The crude product was purified from Petroleum ether/CH_2Cl_2 (10:1 to 1:1, v/v) to give diethyl 2-amino-6-bromoazulene-1,3-dicarboxylate **3** as a brown crystalline solid. Yield 89% (0.89 g). ^{1}H NMR (600 MHz, $CDCl_3$) δ 8.87 (d, J = 11.5 Hz, 2H), 7.83 (d, J = 11.5 Hz, 2H), 4.47 (q, J = 7.1 Hz, 4H), 1.55 (s, 2H), 1.47 (t, J = 7.1 Hz, 6H). ^{13}C NMR (151 MHz, $CDCl_3$) δ 166.41, 162.49, 144.51, 135.51, 129.64, 128.54, 101.13, 60.24, 14.77.

The synthesis of 4 (Diethyl 2-amino-6-(4,4,5,5-tetramethyl-1,3,2-dioxaborolan-2-yl)azulene-1,3-dicarboxylate). In nitrogen atmosphere, diethyl 2-amino-6-bromoazulene-1,3-dicarboxylate **3** (1.00 g, 2.70 mmol, 1.00 eq), [1,1'-bis (diphenylphosphine)ferrocene] palladium dichloride dichloromethane complex (0.10 g, 0.14 mmol, 0.05 eq), potassiumacetate (0.80 g, 8.04 mmol, 3.00 eq) and Bis (pinacol) diboron (0.70 g, 3.00 mmol, 1.1 eq) were combined and 1,4 dioxane (60 mL) was added. The mixture was heated at 95 °C for 18 h and then cooled to room temperature. After dilution with water (100 mL), it was extracted with CH_2Cl_2 (4 × 50 mL). The combined organic extracts were washed with water (200 mL), dried over $MgSO_4$, filtered and concentrated under reduced pressure. The crude product was purified by column chromatography and eluted with Petroleum ether/CH_2Cl_2 (8:1 to 1:1, v/v) to obtain diethyl 2-amino-6-(4,4,5,5-tetramethyl-1,3,2-dioxolan-2-yl)azulene-1,3-dicarboxylate **4**. It is a bright orange solid. Yield 50% (0.60 g). ^{1}H NMR (600 MHz, $CDCl_3$) δ 9.07 (d, J = 10.8 Hz, 2H), 8.04 (d, J = 10.8 Hz, 2H), 7.92 (s, 2H), 4.44 (q, J = 7.1 Hz, 4H), 1.46 (t, J = 7.1 Hz, 6H), 1.38 (s, 12H). ^{13}C NMR (151 MHz, $CDCl_3$) δ 166.64, 163.54, 151.10, 147.43, 138.67, 99.75, 84.57, 83.50, 59.86, 24.94. 14.60.

The synthesis of AZU-OH (Diethyl 2-amino-6-hydroxyazulene-1,3-dicarboxylate). Diethyl 2-amino-6-(4,4,5,5-tetra methyl-1,3,2-dioxaborolan-2-yl)azulene-1,3-dicarboxylate **4** (100 mg, 0.24 mmol, 1.00 eq) was dissolved in THF (10 mL). H_2O_2 solution (50 μL, 0.48 mmol, 2.0 equiv) was added. Stir at room temperature for 24 h to obtain a yellowish solution, and then add water (10 mL). The mixture was extracted with CH_2Cl_2 (2 ×20 mL), and the combined organic extracts were dried with $MgSO_4$ and filtered. The filtrate was concentrated under reduced pressure, and the crude material was purified by silica gel column chromatography and eluted with DCM/CH_3OH (10:1 to 3:1, v/v) to obtain orange powder **AZU-OH**. Yield 70% (0.07 g). ^{1}H NMR (600 MHz, DMSO-d_6) δ 11.05 (s, 1H), 8.99 (d, J = 11.7 Hz, 2H), 7.42 (s, 2H), 7.28 (d, J = 11.7 Hz, 2H), 4.35 (q, J = 7.1 Hz, 4H), 1.37 (t, J = 7.1 Hz, 6H). ^{13}C NMR (151 MHz, DMSO-d_6) δ 166.13, 164.42, 159.07, 140.39, 132.44, 121.90, 99.08, 59.68, 15.00. HRMS (ESI) Calculated for AZU-OH (M+Na^+) $C_{16}H_{17}NNaO_5^+$ 326.1004, Found: 326.1003.

The synthesis of AZU-*β* (Diethyl 6-acetoxy-2-aminoazulene-1,3-dicarboxylate). AZU-OH (1.00 g, 3.30 mmol) was dissolved in acetic anhydride, heated at 55 °C without reflux for 2 h, the mixture was extracted with CH_2Cl_2 (2 × 20 mL), and the combined organic extracts were dried with $MgSO_4$ and filtered. The crude material was purified by silica gel column chromatography and DCM to obtain orange powder **AZU-*β***. Yield 40% (400 mg). ^{1}H NMR (600 MHz, $CDCl_3$) δ 9.11 (d, J = 11.6 Hz, 2H), 7.76 (s, 2H), 7.30 (d, J = 11.6 Hz, 2H), 4.46 (q, J = 7.1 Hz, 4H), 2.35 (s, 3H), 1.47 (t, J = 7.1 Hz, 6H). ^{13}C NMR (151 MHz, $CDCl_3$) δ 168.64, 165.40, 160.97, 151.73, 143.09, 128.49, 125.48, 99.77, 58.96, 20.13, 13.62. HRMS (ESI) Calculated for **AZU-*β*** (M+Na^+) $C_{18}H_{19}NNaO_6^+$ 328.1110, Found: 326.1119.

3. The HRMS spectrum of AZU-*β* for CEs activity

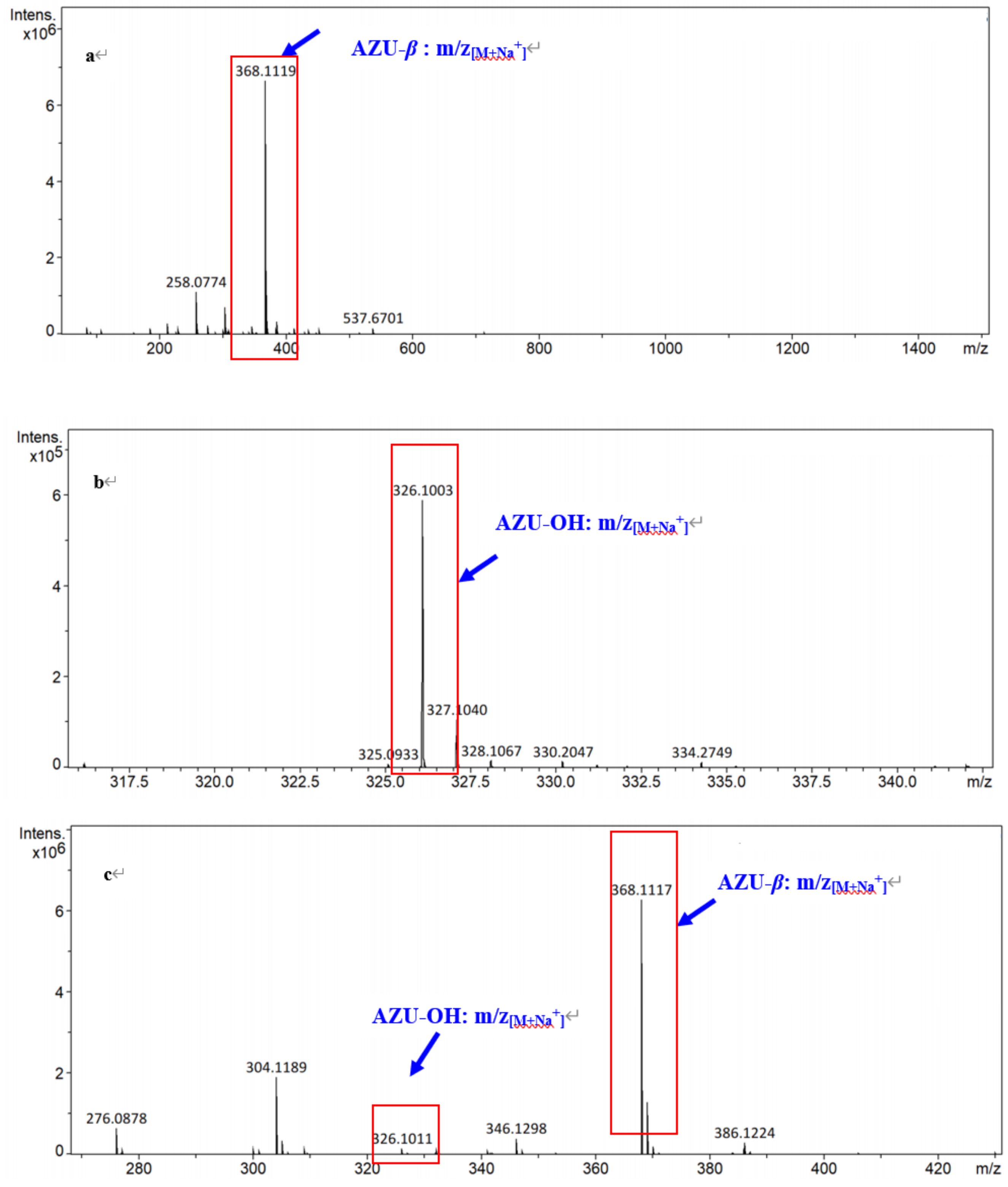

Figure S1. The HRMS spectra of **AZU-*β*** response to CEs. (a), The HRMS spectra of **AZU-*β*** before reaction with CEs; (b), The HRMS spectra of metabolite **AZU-OH**; (c), The HRMS spectra of **AZU-*β*** reacting with CEs.

4. The biocompatibility of AZU-*β*

(1) The selectivity of AZU-*β* for CEs.

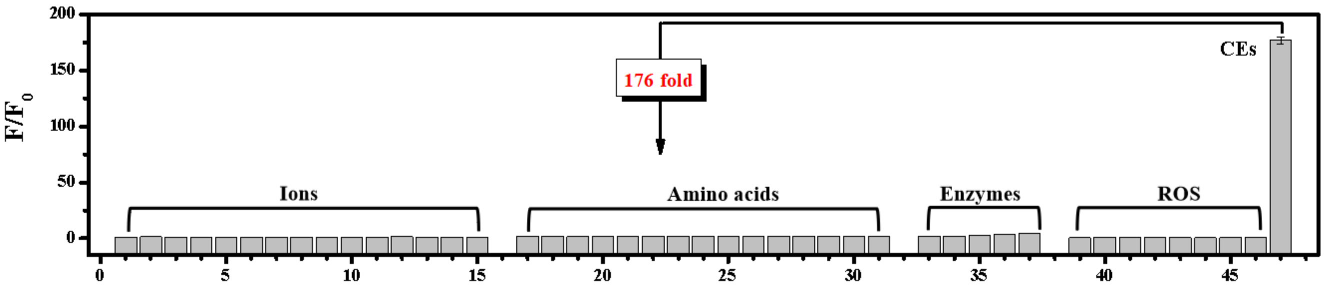

Figure S2. Selectivity of **AZU-*β*** for CEs. Ion interference: 1, $MnSO_4$; 2, Li_2CO_3; 3, $AgNO_3$; 4, $Zn(NO_3)_2$; 5, $MgSO_4$; 6, $CuSO_4$; 7, $FeCl_2$ $4H_2O$; 8, $SnCl_2$; 9, $NaHSO_4$; 10, $CaCl_2$ 6 H_2O; 11, $COCl_2$; 12, $FeCl_3$ $6H_2O$; 13, $CdCl_2$; 14, KCl; 15, NaCl. Amino acid interference: 17, serine; 18, lysine; 19, Glutamine; 20, DL- homocysteine; 21, dithiothreitol acid; 22, glycine -L- complex acid; 23, D- cysteine; 24, Glutamate; 25, glycine; 26, DL- threonine; 27, Cystine; 28, arginine; 29, DL- leucine; 30, l-aspartic acid; 31, Hypoxanthine. Enzymes interference: 33, lysozyme; 34, *β* -amylase; 35, trypsin; 36, pepsin; 37, protease K. ROS interference. 39, $O_2^{\cdot-}$; 40, $NO^{\cdot}$; 41, H_2O_2; 42, $TBO^{\cdot}$; 43, NaClO; 44, $^1O_2$; 45, TBHP; 46, $OH^{\cdot}$. Contrast: 47, CEs (1.0 U/mL). Data are representative of replicate experiments (n = 3).

(2) The pH-stability and the photostability of AZU-*β*.

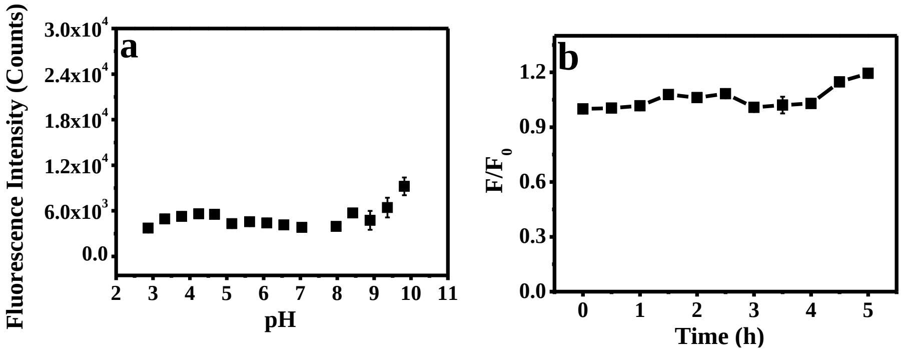

Figure S3. The pH-stability of **AZU-*β*** (a) and the photostability of **AZU-*β*** (b).

(3) The water solubility of AZU-*β* and the metabolites.

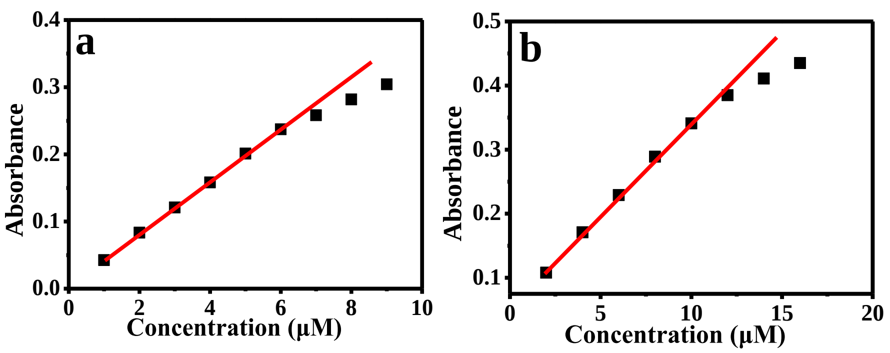

Figure S4. Water solubility of **AZU-*β*** (a) and **AZU-OH** (b).

(4) The cytotoxicity of AZU-*β* and the metabolites.

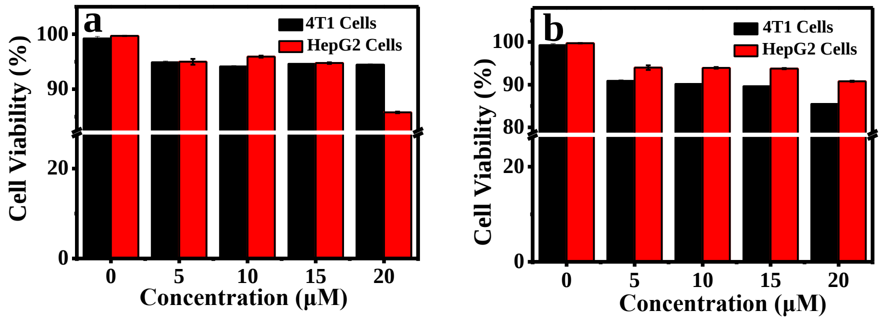

Figure S5. Cytotoxicity of **AZU-*β*** (a) and **AZU-OH** (b).

5. The fluorescence imaging of AZU-*β* in bacteria.

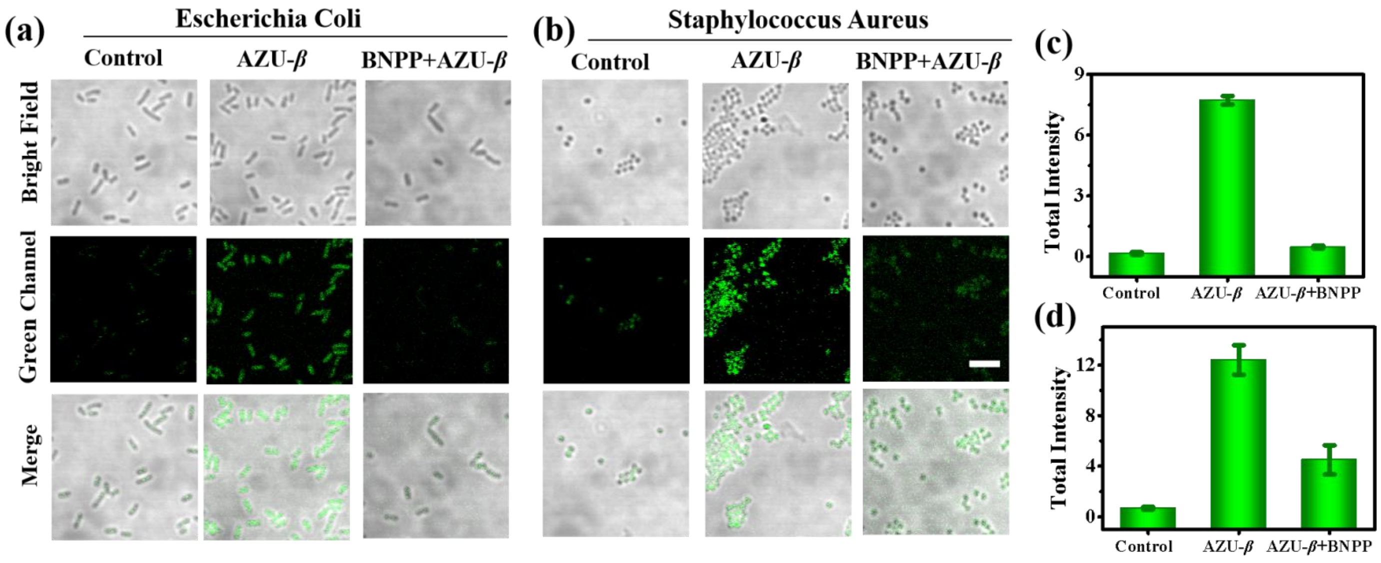

Figure S6. Two-photon fluorescence imaging of **AZU-*β*** (20 μM) in bacteria (*Escherichia coli* and *Staphylococcus aureus*). a and b, Fluorescence imaging in *Escherichia coli* (a) and *Staphylococcus aureus* (b). c and d, The total intensity data for green channels in a (c) and b (d). Control group: Bacteria were not treated; **AZU-*β*** group: Bacteria were incubated with **AZU-*β***; BNPP+**AZU-*β*** group: Bacteria were incubated with BNPP (200 μM) for 30 min, and then incubated with **AZU-*β***. Two-photon excitation wavelength = 800 nm, scan range of Green Channel = 495-540 nm. Internal PMTs are at 16 bit and 1600×1600 pixels, and scan speed is 400 Hz. Scale bar: 40 μm.

6 Appendix

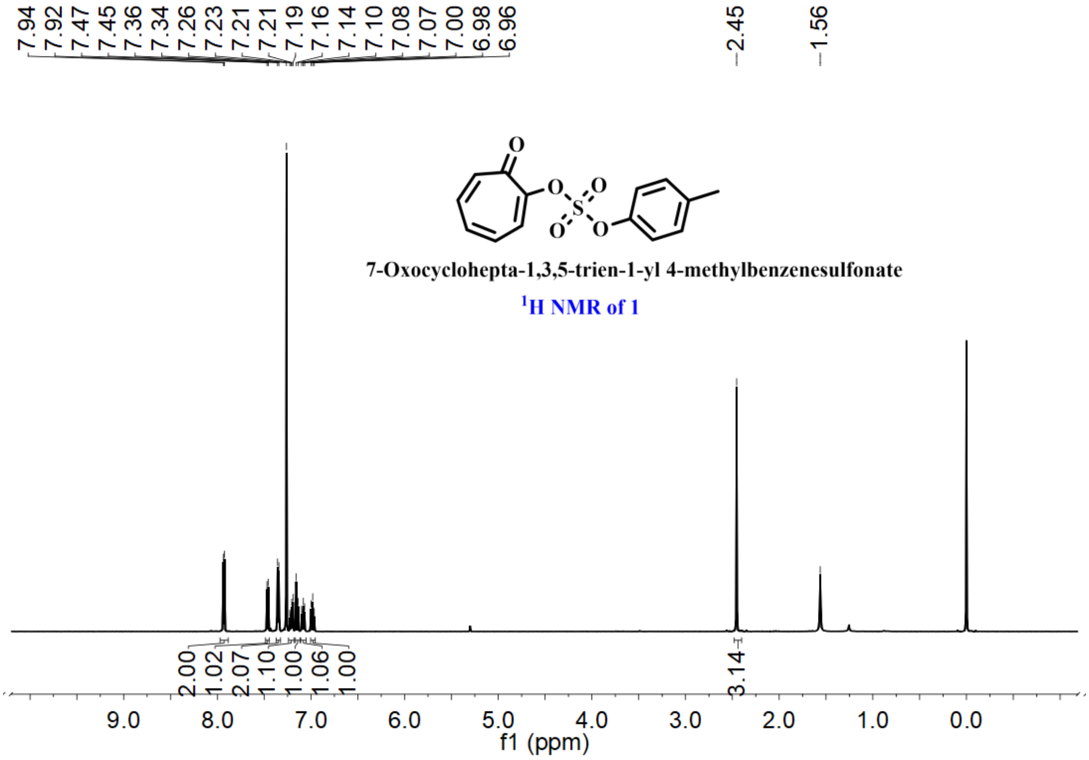

Figure S7. ^{1}H NMR spectrum of **compound 1**.

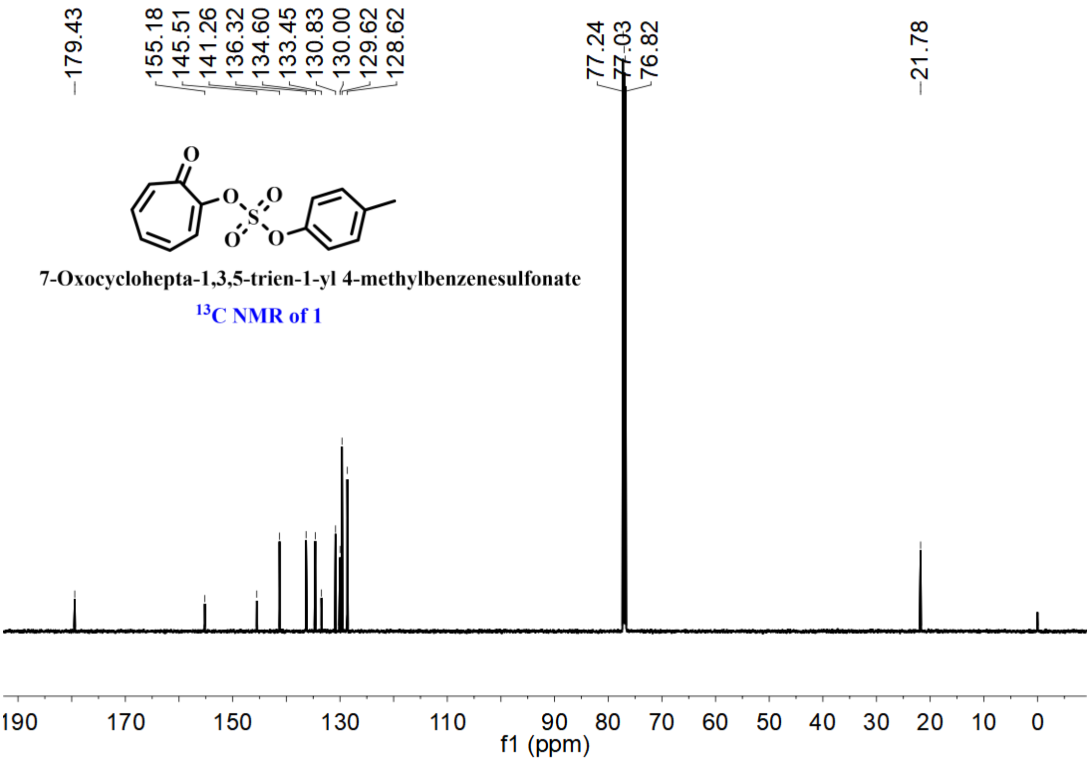

Figure S8. ^{13}C NMR spectrum of **compound 1**.

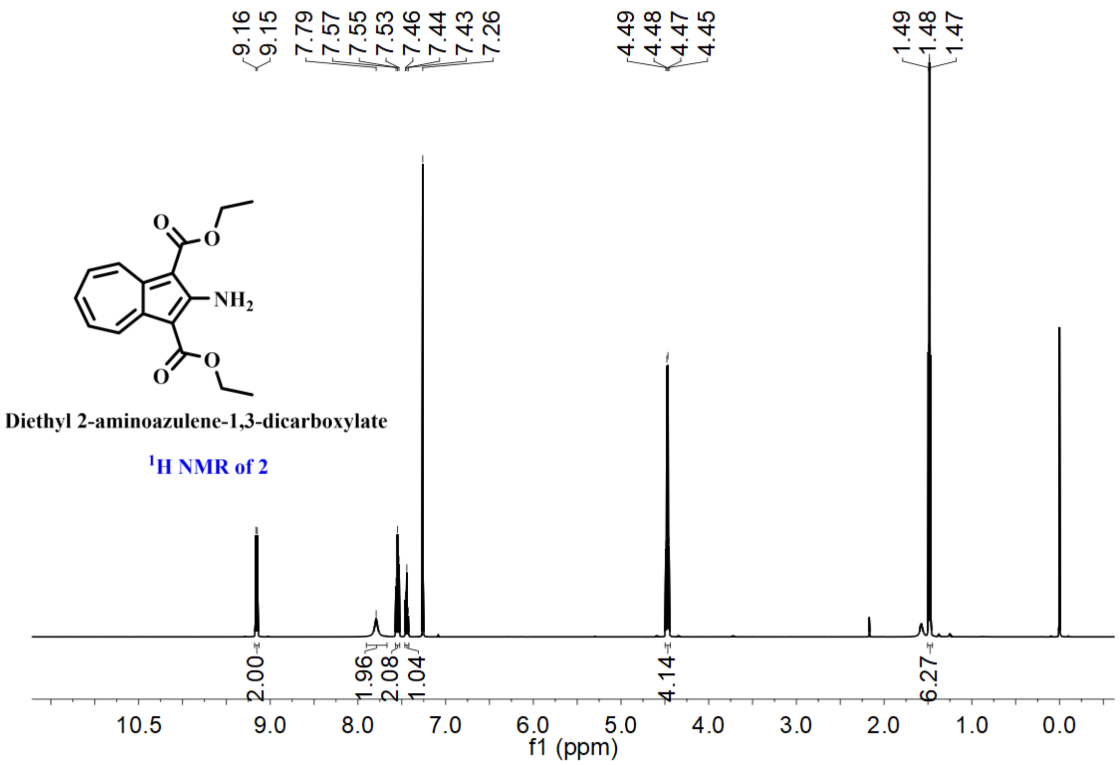

Figure S9. ^{1}H NMR spectrum of **compound 2**.

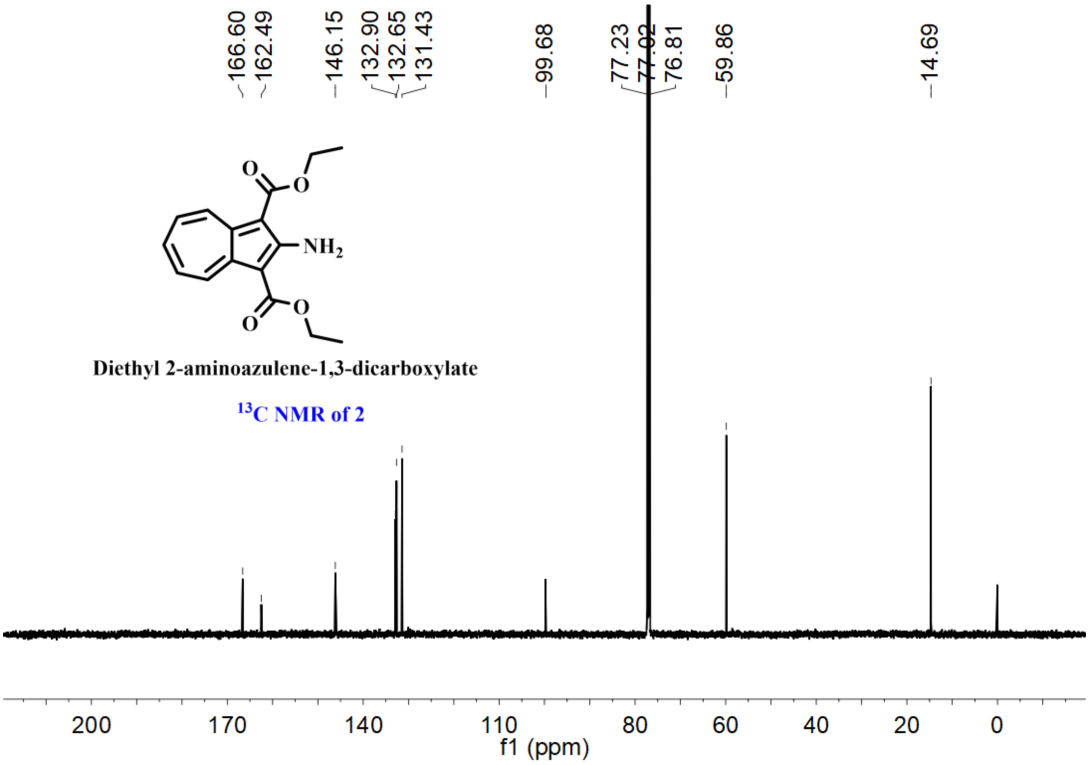

Figure S10. ^{13}C NMR spectrum of **compound 2**.

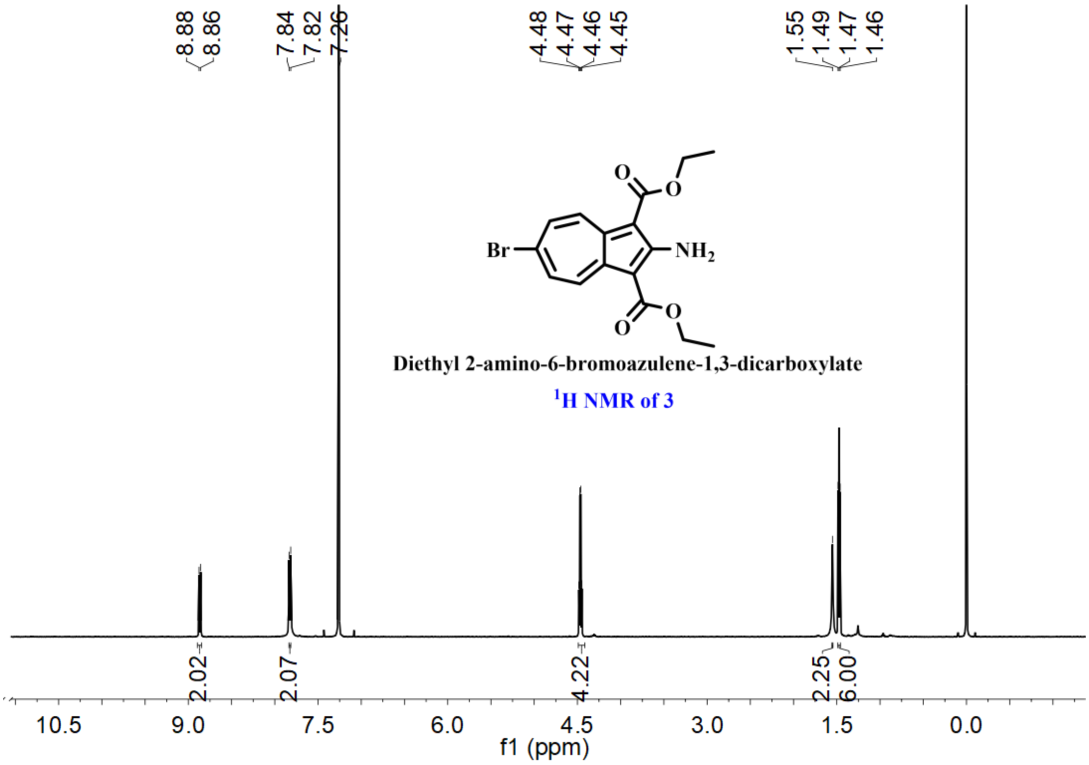

Figure S11. ^{1}H NMR spectrum of **compound 3**.

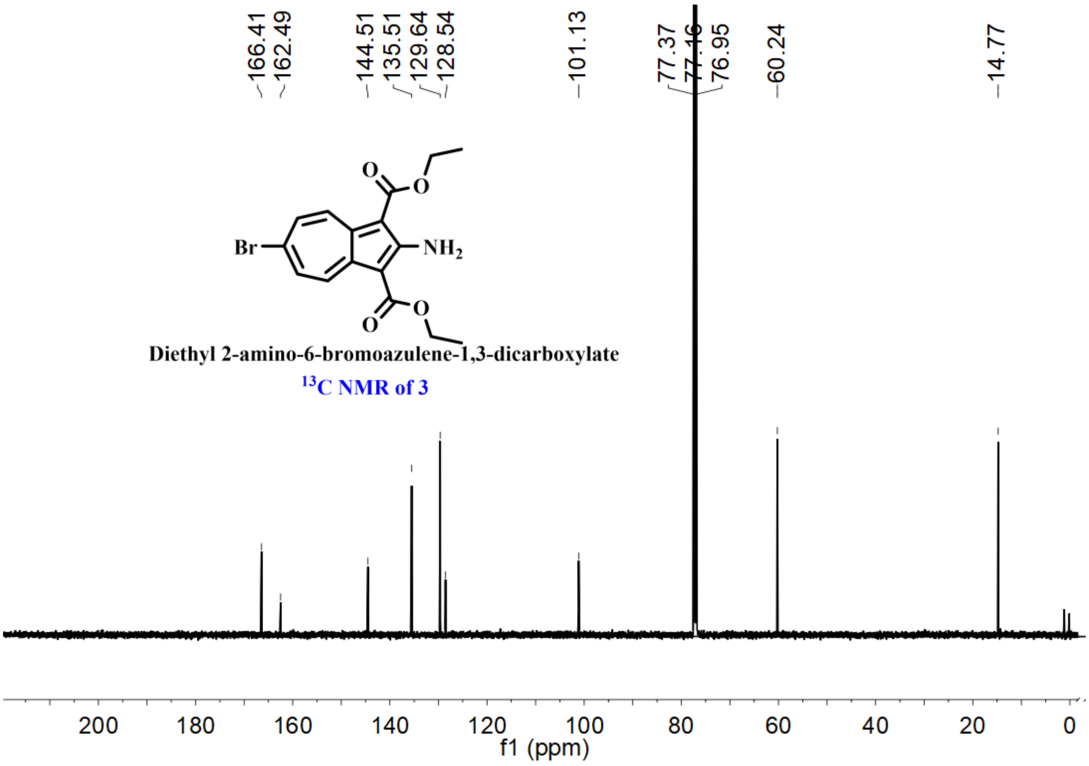

Figure S12. ^{13}C NMR spectrum of **compound 3**.

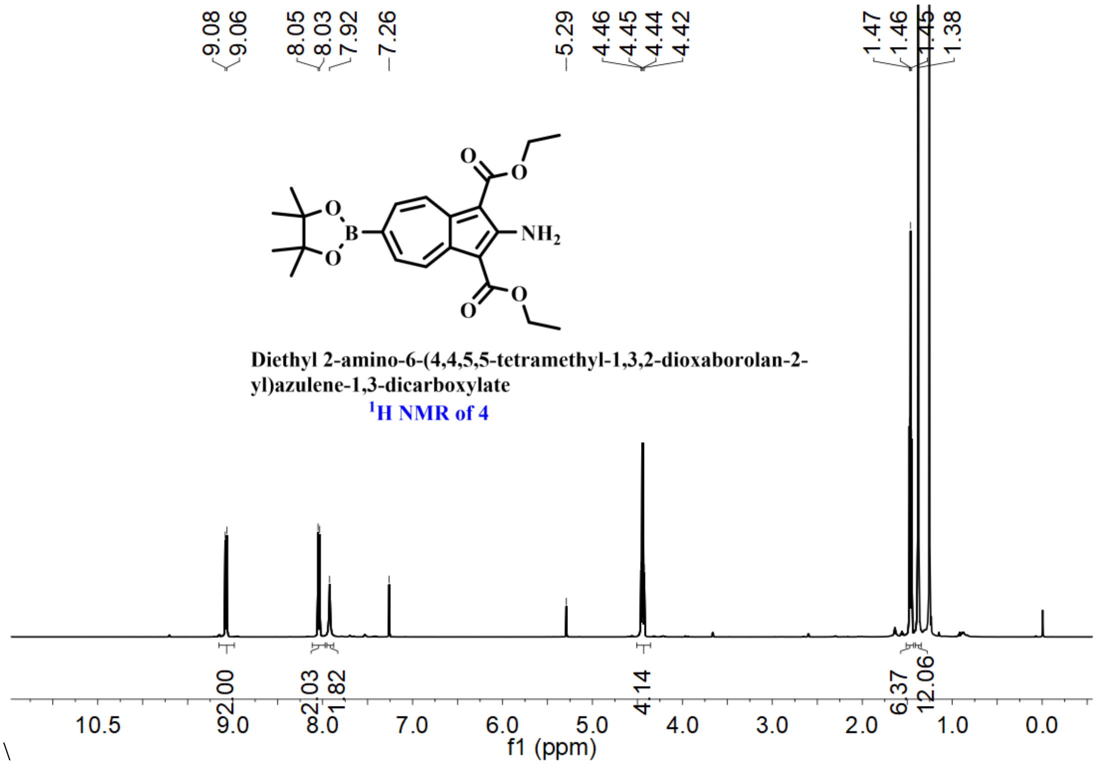

\

Figure S13. ^{1}H NMR spectrum of **compound 4**.

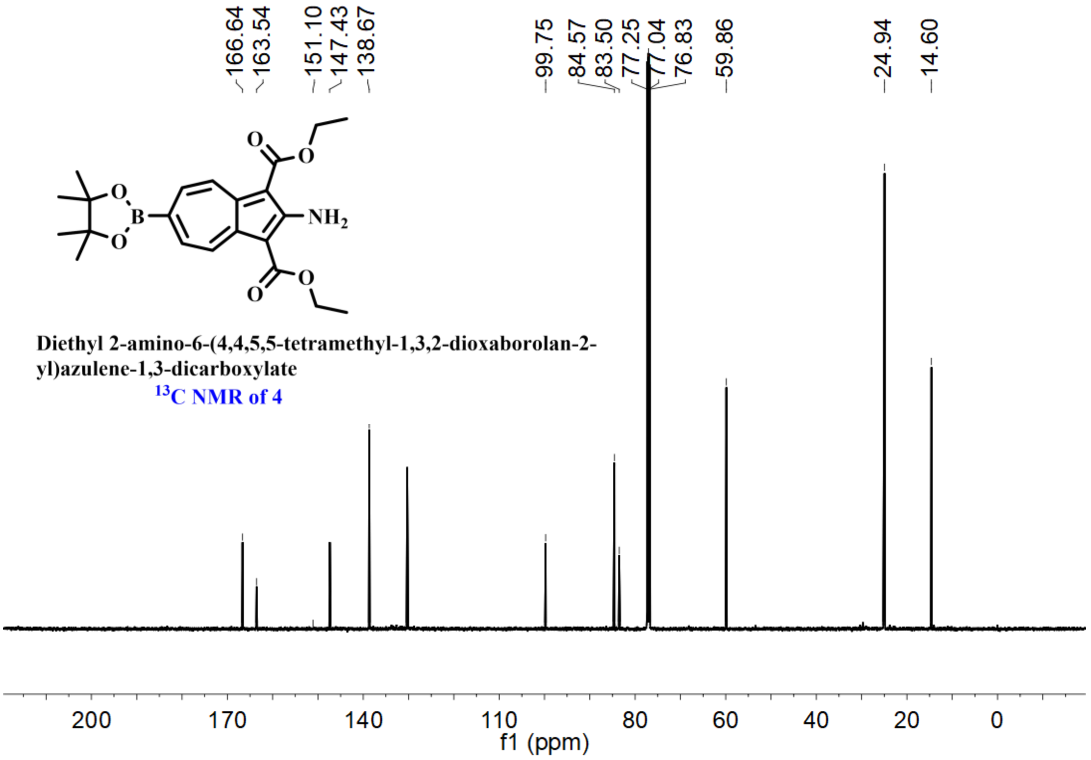

Figure S14. ^{13}C NMR spectrum of **compound 4**.

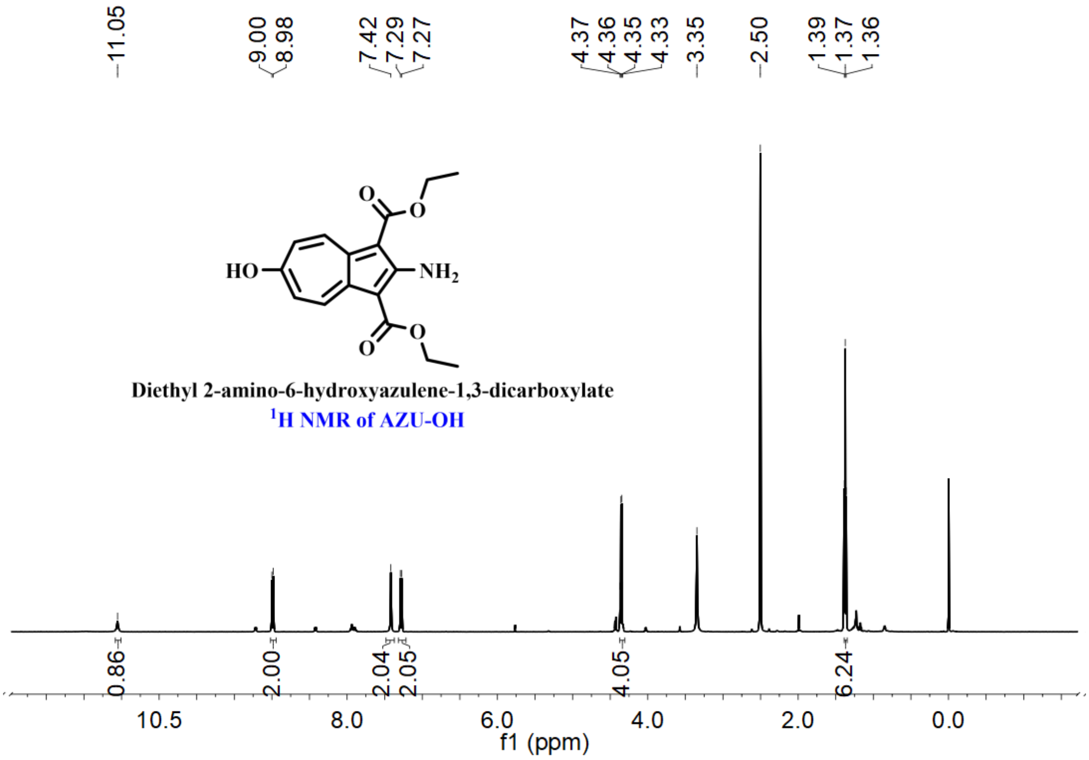

Figure S15. ^{1}H NMR spectrum of **AZU-OH**.

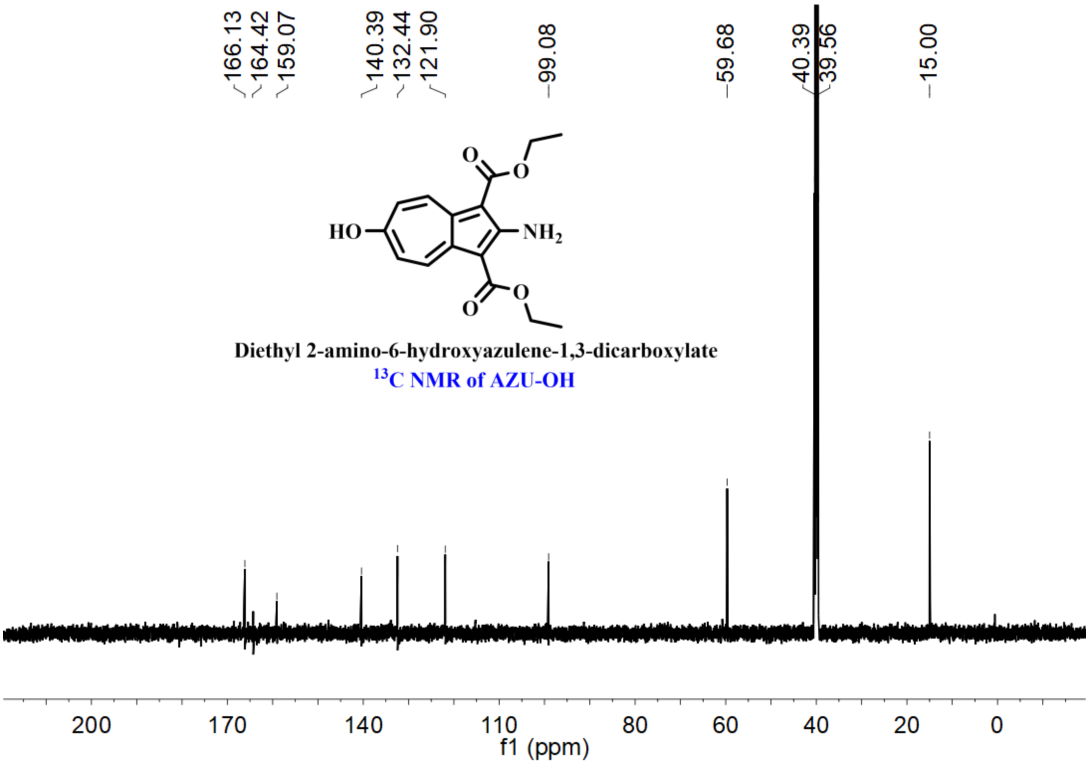

Figure S16. ^{13}C NMR spectrum of **AZU-OH**.

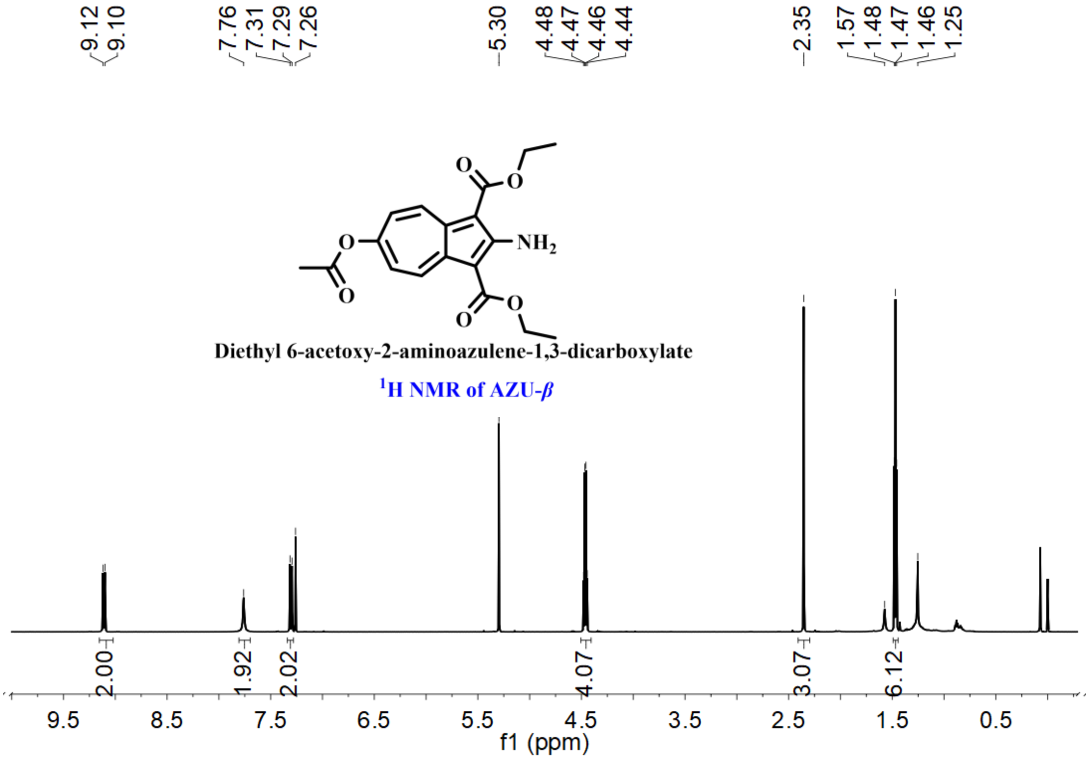

Figure S17. ^{1}H NMR spectrum of **AZU-*β***.

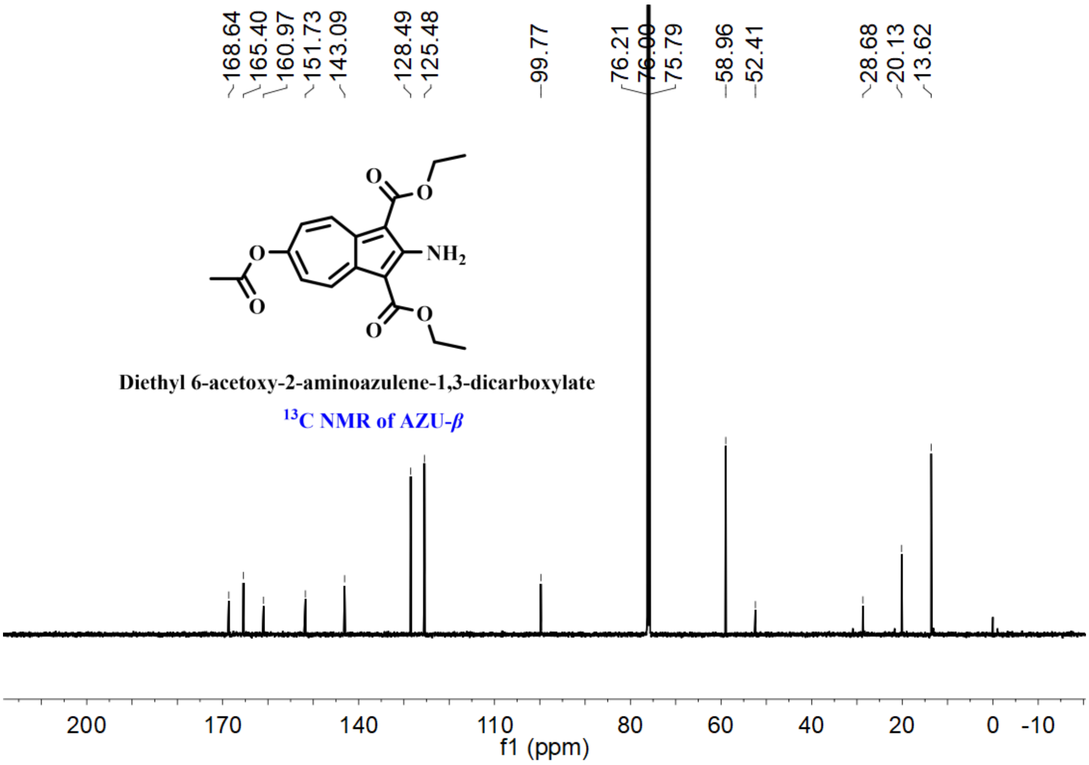

Figure S18. ^{13}C NMR spectrum of **AZU-*β***.
